# Ursolic acid induces colorectal cancer cells ferroptosis via regulation of system xc^-^ and miR-214-3p/Stat3/GPX4 axis

**DOI:** 10.3389/fimmu.2025.1674321

**Published:** 2025-11-18

**Authors:** Yao Shi, Changju Ma, Xiaojuan Tang, Haojie Su, Yue Lu, Jianan Wei, Li Li, Tianhua Huang, Xicheng Wang, Xintian Qin, Ying Ding, Ling Han, Jingjing Wu

**Affiliations:** 1 The Second Clinical College of Guangzhou University of Chinese Medicine, Guangdong Provincial Hospital of Chinese Medicine, Guangzhou, China; 2 Department of Oncology, Cancer Research Institute of Integrated Traditional Chinese and Western Medicine, the First Affiliated Hospital of Guangdong Pharmaceutical University, Guangzhou, China; 3 Central Laboratory, Hunan Provincial Hospital of Integrated Traditional Chinese and Western Medicine, The Affiliated Hospital of Hunan Academy of Traditional Chinese Medicine, Changsha, China; 4 State Key Laboratory of Dampness Syndrome of Chinese Medicine, The Second Affiliated Hospital of Guangzhou University of Chinese Medicine, Guangzhou, China; 5 Guangdong Provincial Key Laboratory of Clinical Research on Traditional Chinese Medicine Syndrome, Guangzhou, China

**Keywords:** ursolic acid, ferroptosis, GPX4, stat3, MiR-214-3p, colorectal cancer

## Abstract

**Background and aim:**

Colorectal cancer (CRC) is a prominent worldwide health concern because of its high prevalence and mortality rates. This study explored the role of Ursolic Acid (UA) in preventing the development of CRC and clarified potential mechanisms.

**Experimental procedure:**

Differential genes between human normal tissues and colon adenocarcinoma tumor tissues, plus survival analysis were generated on GEPIA2 website. RNA-seq was utilized to screen therapeutic targets after UA added into HT29 cells. AutoDock Vina 1.2.3 was used to carry out molecular docking between GPX4 and UA. Dual-luciferase reporter method was applied to evaluate miR-214-3p/GPX4 and miR-214-3p/Stat3 sponging. Gene overexpression plasmids, miRNA mimics and inhibitors transfection assays were carried out. The morphological alterations in mitochondria were detected based on transmission electron microscopy (TEM). *In vivo*, the xenograft model of HT29 cells transfected with luciferase gene (HT29-luc) was constructed in nude mice.

**Key results:**

We found that UA substantially inhibited the proliferation of CRC cells, induced cellular ferroptosis by decreasing the expression of system xc^-^ (SLC7A11 and SLC3A2) and GPX4. Overexpression of Stat3 increased GPX4 expression level. MiR-214-3p mimics can reduce GPX4, p-Stat3 and Stat3 expression levels. MiR-214-3p can bind both GPX4 and Stat3 mRNA 3’UTR. Overexpression of GPX4 and miR-214-3p inhibitors accelerated CRC cells proliferation. MiR-214-3p inhibitors can reverse UA-reduced GPX4 and Stat3 mRNA expression levels. TEM images showed that mitochondrial volume decreased, bilayer membrane density increased, mitochondrial cristae decreased after intervention with UA or miR-214-3p mimics. According to *in vivo* experiments, UA inhibited CRC tumor growth by regulation of above genes.

**Conclusions and implications:**

This study demonstrated that UA could effectively inhibit CRC proliferation by inducing ferroptosis via regulation of system xc^-^ subunits and miR-214-3p/Stat3/GPX4 axis, suggesting UA could serve as a promising anti-colorectal cancer candidate requiring further validation and optimization.

## Introduction

Morbidity (9.6%) and mortality (9.3%) rates of colorectal cancer (CRC) rank third and second among all cancers globally in the year 2022, respectively (1). Patients diagnosed with early-stage CRC may achieve a cure through surgical resection; however, those with advanced-stage CRC typically require chemotherapy. Regrettably, a significant proportion of newly diagnosed CRC patients present with distant metastases at the time of diagnosis (2). Moreover, majority of chemotherapeutic agents can trigger inevitable drug side effects (3). Therefore, there exists an urgent necessity to investigate the mechanisms underlying the pathological development of CRC, with the aim of identifying more effective and safer anti-cancer therapeutics.

*Hedyotis diffusa* Willd (HDW) is a member of the Rubiaceae family. This herb has a rich history of use in traditional Chinese medicine, spanning thousands of years, primarily for its heat-clearing, detoxifying, and blood stagnation alleviation properties (4). Numerous studies substantiate its efficacy as an anti-tumor agent across various cancer types, including, but not limited to, colorectal, liver, breast, and ovarian cancers (5). Ursolic acid (UA), a pentacyclic triterpenoid extracted from HDW, has been confirmed through HPLC analysis to constitute approximately 4% of the herb’s composition (6). UA has demonstrated promising therapeutic effects, including the induction of anticancer, antioxidant, anti-inflammatory properties (7). Previous research has indicated that UA promoted autophagy, apoptosis in gemcitabine-resistant breast cancer cells (8). Additional findings indicate that UA inhibits cholesterol biosynthesis and exerts a suppressive effect on the growth of hepatocellular carcinoma cells (9). Another study has concluded that UA repressed breast cancer cells stemness and progression via modulating Argonaute-2 (10). Nonetheless, the applicability of UA in the treatment of colorectal cancer (CRC) cell progression has yet to be thoroughly explored.

Ferroptosis represents a unique form of regulated cell death (RCD) characterized by the lethal accumulation of lipid peroxides associated with iron. Cells undergoing ferroptosis exhibit distinct features when compared to other extensively studied types of RCD, such as apoptosis, necroptosis, and pyroptosis (11). Recently, regulating ferroptosis to impact cancer progression has been a hotspot. Ferroptosis plays crucial role in tumor biology and tumor immunity. Inducing ferroptosis was found to be a promising strategy to overcome resistance to immune checkpoint inhibitors (12). Furthermore, ferroptosis has been identified as a significant target for therapeutic strategies in colorectal cancer (CRC) (13). Currently, four categories of markers have been established as reliable indicators for detecting ferroptosis, which include lipid peroxidation, the re-localization of the transferrin receptor (TFR1), changes in mitochondrial morphology, and alterations in gene expression.

In this research, comprehensive mechanistic investigation integrating RNA-seq, molecular docking, dual-luciferase assays, and *in vivo* validation were utilized. We clarified that UA exhibits obvious anti-tumor capacity in CRC cells by promoting ferroptosis via modulation of system xc^-^ and miR-214-3p/Stat3/GPX4 axis, which eventually depletes glutathione (GSH), inactivates GPX4 and decreases GPX4 expression levels. Additionally, UA effectively and safely induces cellular ferroptosis in tumor sample. Identification of the miR-214-3p/Stat3/GPX4 regulatory axis adds novel mechanistic insight into CRC ferroptosis biology. Therefore, the obtained findings provide a promising therapeutic measure for tackling CRC progression.

## Materials and methods

### Reagents

Ursolic Acid was bought from the Chengdu Must Enterprise (Chengdu, China) and was diluted before use. The relevant SYBR qPCR Mix, cDNA Synthesis Kit were bought from Vazyme (Nanjing, China). The corresponding Protein Marker was from YEASEN (Shanghai, China). Erastin, ferrostatin-1, and Z-VAD-FMK were from MedChemExpress (HY-15763, HY-100579 and HY-16658B, USA). Trizol and Lipofectamine 3000 reagent were bought from Thermo Fisher Scientific. GPX4 and GAPDH antibodies were purchased from Abcam, phospho-Stat3 (Tyr705), Stat3, and SLC3A2 antibodies from Cell Signaling Technology Lnc (MA, USA). SLC7A11 antibody was from Boster Biological Technology Co. Itd (Wuhan, Hubei, China). The EdU cell proliferation kit, inhibitors and mimics of miR-214-3p, PCR primers were bought from RIBOBIO (Guangzhou, China).

### Cell culture

HT29, Caco2 and HCT116 CRC cells were obtained from Chinese Academy of Sciences Cell Bank. All cells were cultured at 37°C, in a standard atmosphere with DMEM medium (Gibco, USA) with 10% FBS and 1% penicillin-streptomycin solution (Gibco, USA).

### Cell viability assay

Viability was assessed based on the MTT method. In the experimental process, cells were cultured with density of 5×10^3^/plate. And UA was added to intervene cells for up to 72 hr. Regarding separate experiments, the LV105-GPX4 plasmids were also transfected into HT29 cells 24 hr before being exposed to UA for an additional 24 hr. Then, 10μL MTT solution (5g/L) was added for another 4 hr. Subsequently, the formazan was dissolved with DMSO, and OD was detected on microplate reader.

### RNA-seq

Transcriptome analysis was performed on UA-treated and untreated HT29 cells with three replicates. Cell samples were sent to Metware Co., Ltd. (Wuhan, China) for RNA-Seq analysis. DESeq2 software was used to analyze differentially expressed genes (DEGs), and *P* value was corrected via Benjamini-Hochberg method. |log2FoldChange|≥1 was used as the threshold to be considered as significant differential expression. The enrich KEGG algorithms was used to KEGG analysis on DEGs. The raw sequences data reported in this research was deposited in public repository NCBI SRA with accession number PRJNA1344781.

### Colony formation detection

CRC cells were put in 6-well dishes and incubated in mixture with UA (20 μM) for 10 days, and media was changed every three days. The cells were stained for 0.5h with crystal violet. Colonies were detected via microscope (Nikon, Japan).

### Cell apoptosis assays

Cell apoptosis assays were undertaken with the Annexin V-FITC/PI kit (MULTI SCIENCES, China) as previously reported (14). After administration by UA or erastin, cells were collected, centrifuged, then resuspended in binding buffer. Next, Annexin V-FITC and PI were added. The samples were analyzed under a flow cytometer (Novo Quanteon, USA).

### GSH assay, Fe^2+^ assay and MDA assay

GSH assay kit (BC1175), Fe^2+^ assay kit (BC5315) and MDA assay kit (BC0025) were bought from Beijing Solarbio Enterprise. The standards and samples were prepared according to manufacturer’s instruction, then the optical density value was measured at 412 nm (GSH assay), 510 nm (Fe^2+^ assay), 532nm and 600 nm (MDA assay).

### Detection of ROS and lipid peroxidation

To detect the ROS level, the HT29 and HCT116 cells were marked via DCFH-DA fluorescent probe (MCE, USA). In the experiment, cells were treated with UA and stained via DCFH-DA (10μM) for 0.5 hr, then washed by PBS. The fluorescence signal of it was detected based on a flow cytometer (Novo Quanteon, USA). The relevant C11-BODIPY fluorescent probe (Thermo Fisher Scientific, USA) was applied to detect peroxidation. Cells were cultured with 5 μM C11-BODIPY fluorescent probe at 37 °C for half an hour, then detected via microscopy (ZEISS LSM710, Germany).

### Molecular docking

AutoDock Vina 1.2.3 software was used to calculate the GPX4 and UA docking, and the conformation with low binding energy (high affinity) was derived and input into PyMol 2.5.5 for visualization.

### Cell transfection

Transfection experiment was carried out as in the prior report (15). Mimics of miR-214-3p (100 nM) and inhibitors of miR-214-3p (150 nM) were utilized. The control and overexpression vectors LV105-GPX4 (0.8 μg/ml), pcmv6-Stat3 (0.8 μg/ml) were transfected for 24 hr, then treated with UA for 24 hr for the subsequent testing.

### Western blot

We determined the expression of SLC7A11, SLC3A2, phosphor-Stat3 (p-Stat3), Stat3 and GPX4 proteins via WB method. The protein lysates were prepared through lysis buffer with a protease inhibitor cocktail. In the experimental process, a BCA kit was applied to detect the protein levels. All samples were treated via electrophoresis and then transferred into the PVDF membrane, then blocked in 5% BSA for 1h and cultured in primary antibodies (SLC7A11, SLC3A2, phosphor-Stat3, Stat3, GPX4 and GAPDH) at 4°C. Next, membranes were rinsed, then the proteins were cultured with secondary goat antibody for 60 min (CST, USA). Subsequently, the luminescence medium (Millipore, USA) was used for chemiluminescence detection.

### qRT-PCR

The PrimeScript™RT Kit (GenePharma Eenterprise, China) was applied to synthesis the first strand cDNA. SYBR Green Mix was applied to perform amplification with the program as follow: 30 sec at 95°C; 40 cycles of 10 sec also at 95°C, 30 sec at 60°C. The relevant sequences were designed as follows:

GPX4 forward: 5′-CGATACGCTGAGTGTGGTTTGC-3′,
GPX4 reverse: 5′-CATTTCCCAGGATGCCCTTG-3′;
SLC7A11 forward: 5′-TCTCCAAAGGAGGTTACCTGC-3′,
SLC7A11 reverse: 5′-AGACTCCCCTCAGTAAAGTGAC-3′;
SLC3A2 forward: 5′-TGAATGAGTTAGAGCCCGAGA-3′,
SLC3A2 reverse: 5′-GTCTTCCGCCACCTTGATCTT-3′;
Stat3 forward: 5′-CAGCAGCTTGACACACGGTA-3′,
Stat3 reverse: 5′-AAACACCAAAGTGGCATGTGA-3′;
GAPDH forward: 5′-CTCCTCCTGTTCGACAGTCAGC-3′,
GAPDH reverse: 5′-CCCAATACGACCAAATCCGTT-3′;
U6 forward: 5′-ATTGGAACGATACAGAGAAGATT-3′,
U6 reverse: 5′-GGAACGCTTCACGAATTTG-3′;
miR-214-3p forward: 5′- CAATACTGACAGCAGGCACA-3′,
miR-214-3p reverse: 5′- TATGGTTGTTCACGACTCCTTCAC-3′.

### Immunofluorescence staining

CRC cells were permeabilized based on Triton X‐100 (Sigma‐Aldrich), incubated with normal goat serum for half an hour, and cultured with primary antibodies against p-Stat3 antibody (1:100) and GPX4 antibody (1:200) at 4 °C for 12 hours. The next day, Alexa Fluor antibodies was added and cultured for 1 h. The relevant quenching sealing agents were added into wells, and then detected via confocal microscopy (ZEISS, Germany).

### The TEM imaging

For imaging mitochondria, HT29 cells were digested, and collected in a 1.5 mL EP tube and centrifuged for subsequent experiment. The cell mass was fixed with 2.5% glutaraldehyde, stored for 12h at 4 °C, and stained via 1% OsO4. All samples were sectioned, subsequently poststained with uranyl acetate for 10 min before detection using JEM-1400 Flash electron microscopy.

### Dual-luciferase reporter assay

The two types Stat3 and GPX4 3’UTR vectors were prepared. Transfected vectors into cells with miR-214-3p mimics. Luciferase activities were tested based on relevant HS Assay Kit (GeneCopoeia, USA).

### Animal experiments

Animal experiments were undertaken based on the instruction and approved by present Hospital Committee (No 2023019). Mice were bought form Beijing Vital River Company. 2 × 10^6^ HT29-luc cells were injected to right flank. After seven days, via generating random numbers in Excel, the mice were randomly divided into four groups (n=7), Control (saline), erastin (10 mg/kg), UA (20 mg/kg), UA (40 mg/kg) groups. Saline, erastin or UA was administered via intraperitoneal injection once every day for 3 weeks. Tumor volumes were recorded based on the formula: volume = (width^2^ × length)/2. Mice body weights were measured every three days. In terms of bioluminescence imaging experiment, 20 mg/kg luciferin was injected to every mouse. Imaging device (Xenogen, Berkeley, USA) was applied to collect the images. After 21 days, mice were euthanized with intraperitoneal injection of 100 mg/kg pentobarbital sodium solution. Then tumor tissues were undergone detection of proteins and miR-214-3p expression levels.

### Statistical analysis

Normally distributed data are expressed as the mean ± standard deviation in the form of scatter dot plot. Data were collected from at least three biological replicates and three technical replicates. The Shapiro–Wilk test was utilized to evaluate data normality. Statistical analyses, encompassing Student’s t-test, one-way analysis of variance, and Tukey multiple comparison tests, were conducted. Statistical significance was established at *p* < 0.05.

## Results

### Effect of UA on CRC cells proliferation

**Figure 1:**
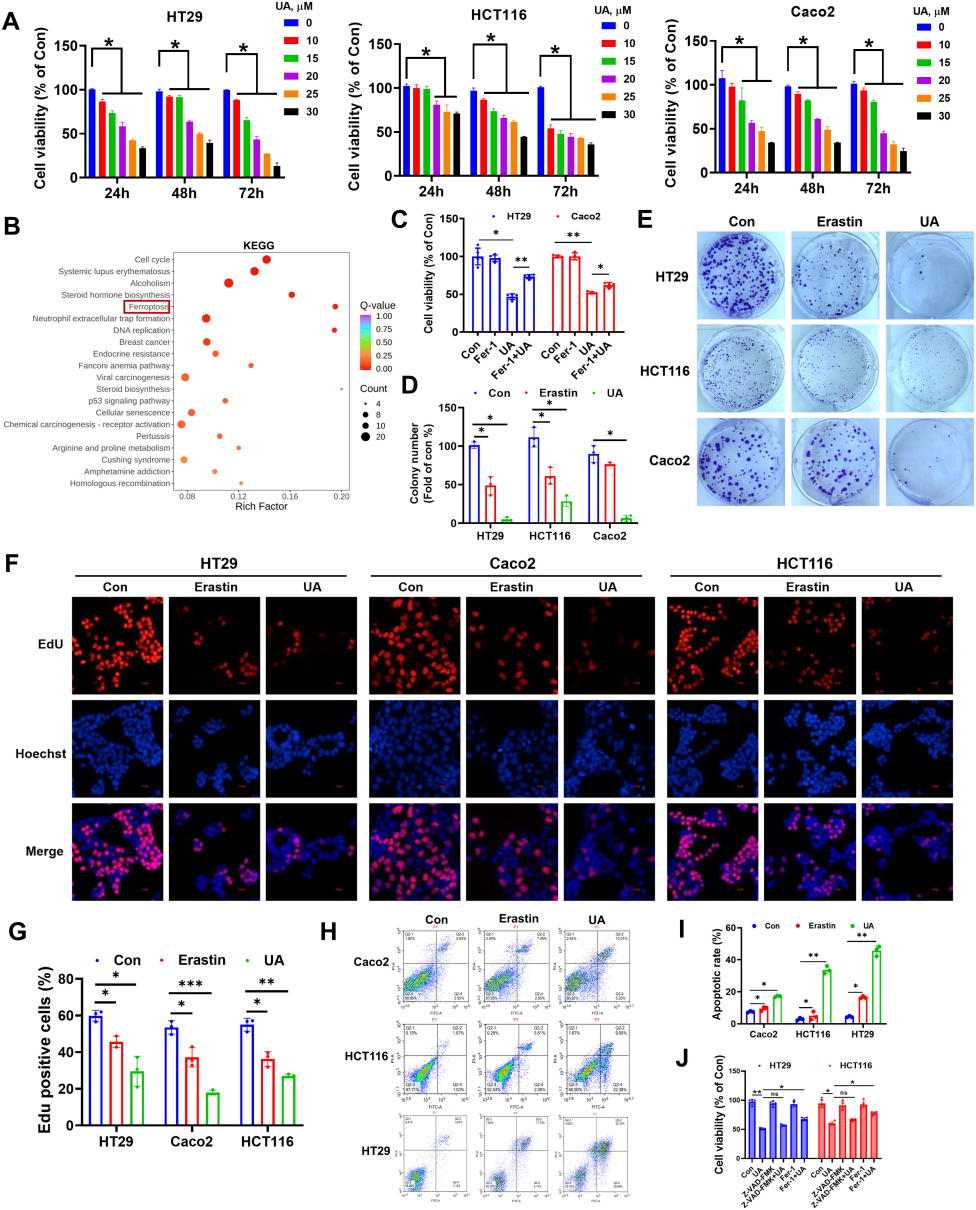
Effect of UA on colorectal cancer cells proliferation. **(A)** Inhibitory effect of different concentrations UA on the cell viability after 24 h, 48 h and 72 h treatment by MTT assays (n=4). **(B)** KEGG enrichment analysis of HT29 cells differentially expressed mRNAs after UA intervention (n=3). **(C)** Cells were treated with UA (20 μM) in the absence or presence of Fer-1 (1 μM) for 24 h, cell viability was tested via MTT assay (n=5). **(D, E)** Colony formation assay to study the long-term effect of UA (20 μM) and erastin (10 μM) on CRC cells (n=3). **(F, G)** EdU assay was applied to determine cell proliferation (n=3). **(H, I)** Annexin V-FITC/PI assays were utilized to analyze cell apoptosis (n=3). **(J)** Cells were treated with UA (20 μM) in the absence or presence of Z-VAD-FMK (10 μM), Fer-1 (1 μM) for 24 h, cell viability was tested via MTT assay (n=5). *p < 0.05, **p < 0.01, ***p < 0.001. ns, not significant.

We chose the colorectal cancer cell lines (HT29, HCT116, Caco2) as the experimental objects. MTT assay showed that UA inhibited CRC cells proliferation (**Figure 1A**). We chose a UA concentration not exceeding 20 μM as standard concentration for subsequent tests. RNA-Seq was performed on HT29 cells after UA intervention. The differentially expressed genes were further subjected to KEGG enrichment analysis, and ferroptosis was found to be one of the key components (**Figure 1B**). Next, we found that ferrostatin-1 (Fer-1), a ferroptosis inhibitor, can reverse the inhibitory effect of UA on CRC cells proliferation (**Figure 1C**). This phenomenon suggests that UA may exert its effect by promoting ferroptosis in CRC cells. Therefore, in the following experiments, we used erastin, a ferroptosis enhancer, as a positive control drug to further research the inhibitory effect and molecular mechanism of UA on CRC cells growth. We then found that UA obviously decreased colonies number in CRC cells (**Figures 1D, E**). The EdU assay once again confirmed that UA can effectively block the proliferation of CRC cells (**Figures 1F, G**). Since ferroptosis is often interconnected with other cell death pathways, including apoptosis (16, 17), therefore we conducted Annexin V-FITC/PI assays. Our results indicated a higher rate of apoptotic cells in response to UA treatment (**Figures 1H, I**). The data show UA induces both ferroptosis and apoptosis. However, it remains unclear which pathway predominantly contributes to the anti-tumor effect. Therefore, we used apoptosis inhibitor Z-VAD-FMK (10 μM) alongside ferroptosis inhibitor Fer-1 (1 μM) to carry out rescue experiments. We found that only Fer-1 can partially reverse the inhibitory effect of UA on CRC cells viability, which suggested ferroptosis was primary mechanism (**Figure 1J**).

### UA activates ferroptosis in CRC cells

**Figure 2:**
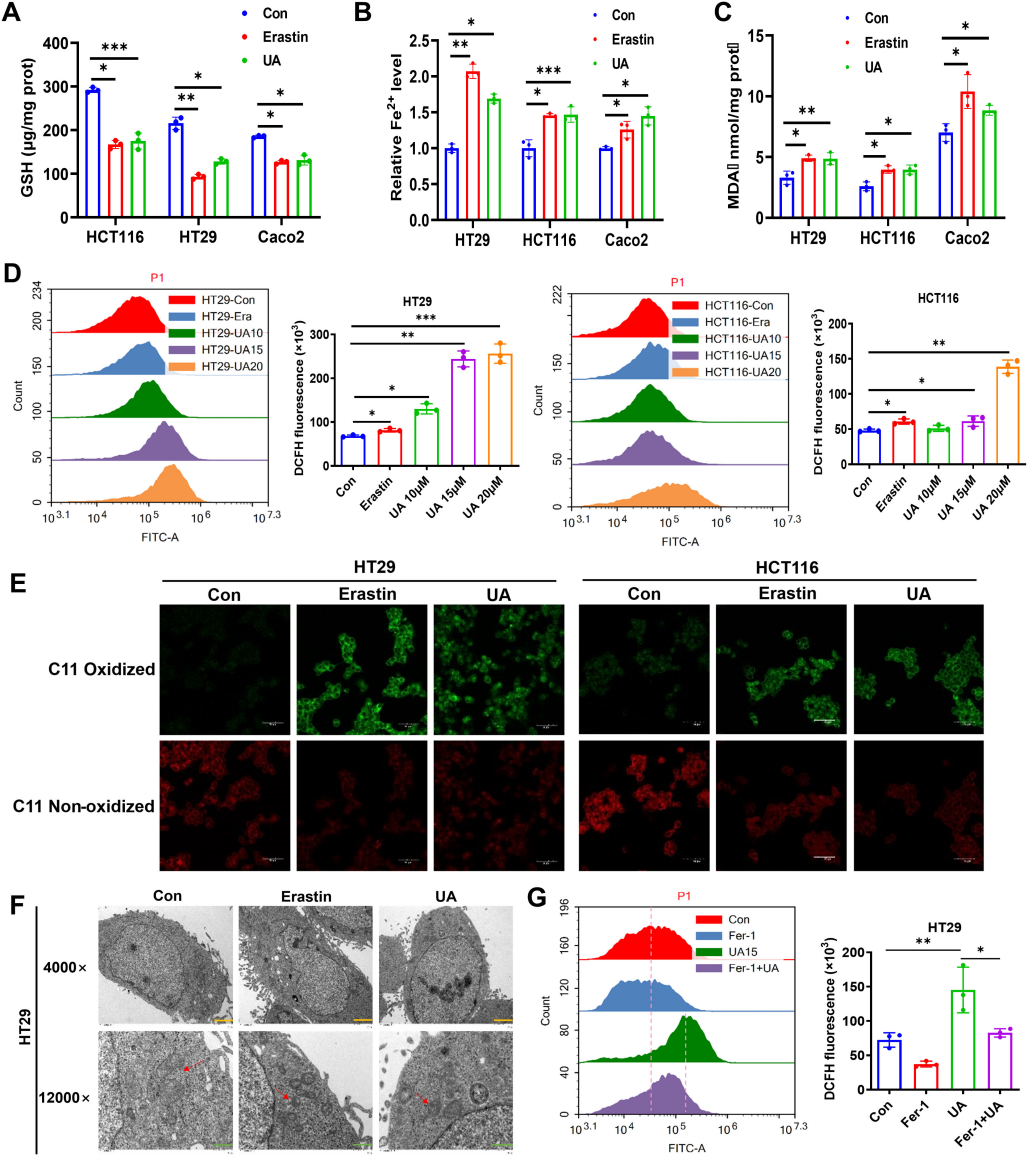
UA induces ferroptosis in colorectal cancer cells. Cells were cultured with or without the presence of UA (20 μM) or erastin (10 μM) for 24 hours. Then, relative levels of **(A)** GSH, **(B)** Fe^2+^ and **(C)** MDA were detected with corresponding reagent kits. **(D)** Analysis of ROS levels in HT29 and HCT116 via flow cytometer. **(E)** Lipid peroxidation was measured using C11-BODIPY fluorescent probe. **(F)** Transmission electron microscope images of mitochondria in HT29 cells with or without drug intervention (Magnification=×4.0k [scale bar, 2 μm] and 12.0k [scale bar, 1 μm]). **(G)** Cells were divided into single drug groups (20 μM UA or 1 μM Fer-1) and a combination drug group. Then ROS levels in HT29 were tested via flow cytometer assays. n=3. *p < 0.05, **p < 0.01, ***p < 0.001.

To further discover the exact influence of UA on CRC cell ferroptosis, we conducted a series of assays. And it was found that GSH levels were reduced (**Figure 2A**), Fe^2+^ and lipid peroxidation products were increased in both erastin (10 μM) and UA (20 μM) treatment group (**Figures 2B, C**). Cellular ROS production was measured, and the data indicated that ROS levels were markedly decreased in UA treatment group (**Figure 2D**). A fluorescent probe was applied to detect the lipid peroxidation. After UA treatment, lipid-ROS levels were remarkably upregulated (**Figure 2E**).

Typical morphological features of ferroptosis contain alterations in mitochondria (18). To further prove the ferroptosis-promoting effect of UA on CRC, the mitochondrial alterations were measured via TEM. The images indicated that, the mitochondrial volume decreased, cristae also decreased after treatment with UA (**Figure 2F**). Moreover, we wondered whether the ROS accumulation after UA treatment was specifically due to ferroptosis, then we added ferroptosis inhibitor, Fer-1. The data showed that Fer-1 can mitigate UA-induced ROS level, which verified our hypothesis (**Figure 2G**).

### UA suppresses system xc^-^ from the level of transcription and translation

**Figure 3:**
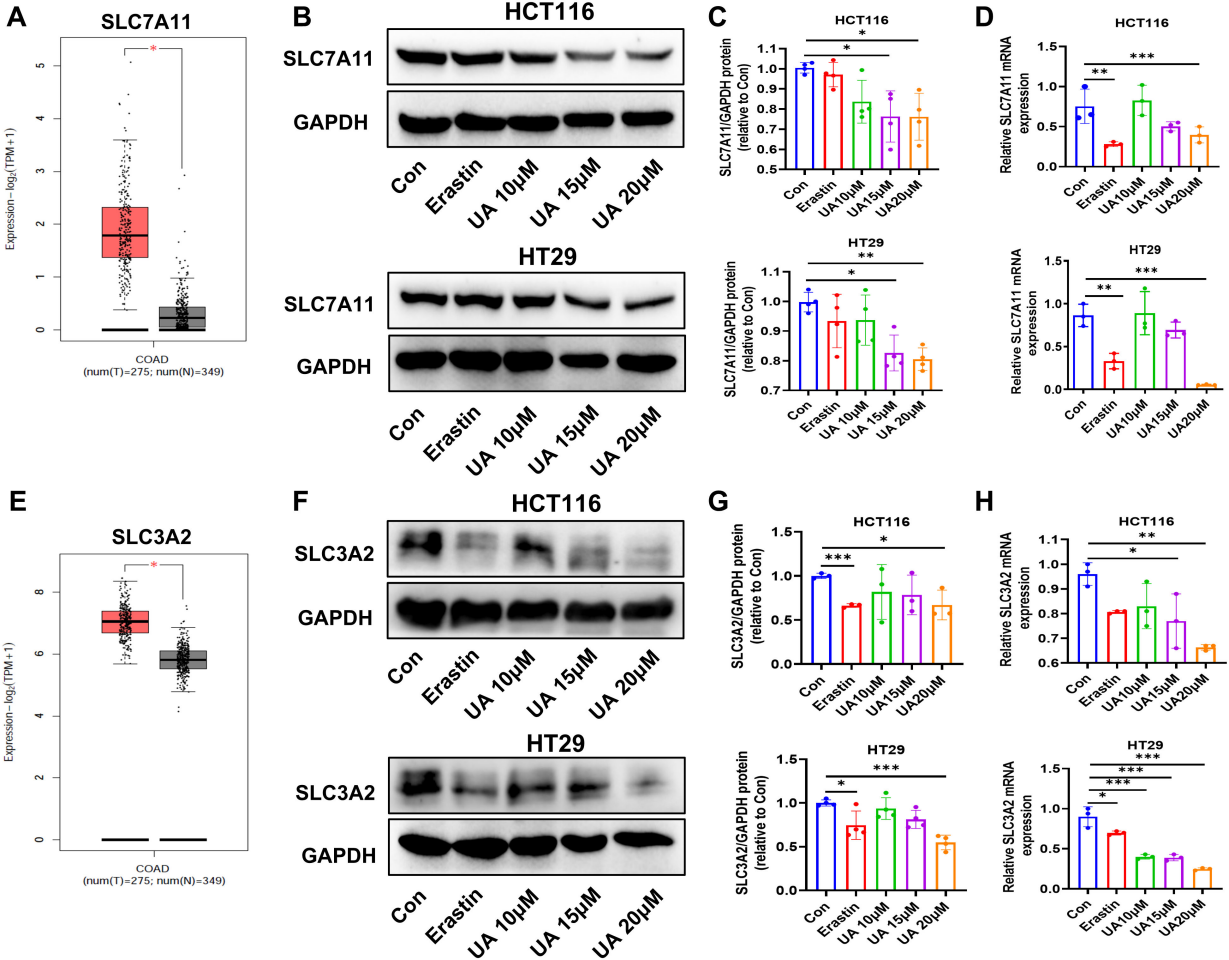
UA reduce SLC7A11, SLC3A2 protein levels and mRNA levels. **(A)** SLC7A11 gene expression in colon adenocarcinoma (COAD) tumor samples, compared with normal samples, searched on http://gepia.cancer-pku.cn/. **(B, C)** SLC7A11 protein expression levels were detected by Western blot after exposure of UA (10 μM, 15 μM, and 20 μM), with erastin (10 μM) as the positive drug (n=4). **(D)** qRT-PCR was used to test mRNA levels of SLC7A11 in HCT116 and HT29 cells (n=3). **(E)** SLC3A2 gene expression in COAD tumor samples and normal samples. **(F, G)** SLC3A2 protein expression levels were detected by Western blot (n=4). **(H)** SLC3A2 mRNA levels was analyzed via qRT-PCR (n=3). *p < 0.05, **p < 0.01, ***p < 0.001.

The system xc^-^ contain SLC7A11 and SLC3A2. They mediate the exchange process of intracellular glutamate. Next, cystine is converted to cysteine, which plays a key role in the process of antioxidant and GSH generation (19, 20). We searched TCGA databases, the results indicated that SLC7A11 expression and SLC3A2 expression were obviously enhanced in colon adenocarcinoma (COAD), compared with normal samples (**Figures 3A, E**). Western blot assays suggested that UA can inhibit SLC7A11 and SLC3A2 protein expression levels (**Figures 3C, G**). The qRT-PCR experiment results showed that UA can repress SLC7A11 and SLC3A2 mRNA expression levels (**Figures 3D, H**).

### UA restrains Stat3/GPX4 regulatory axis

**Figure 4:**
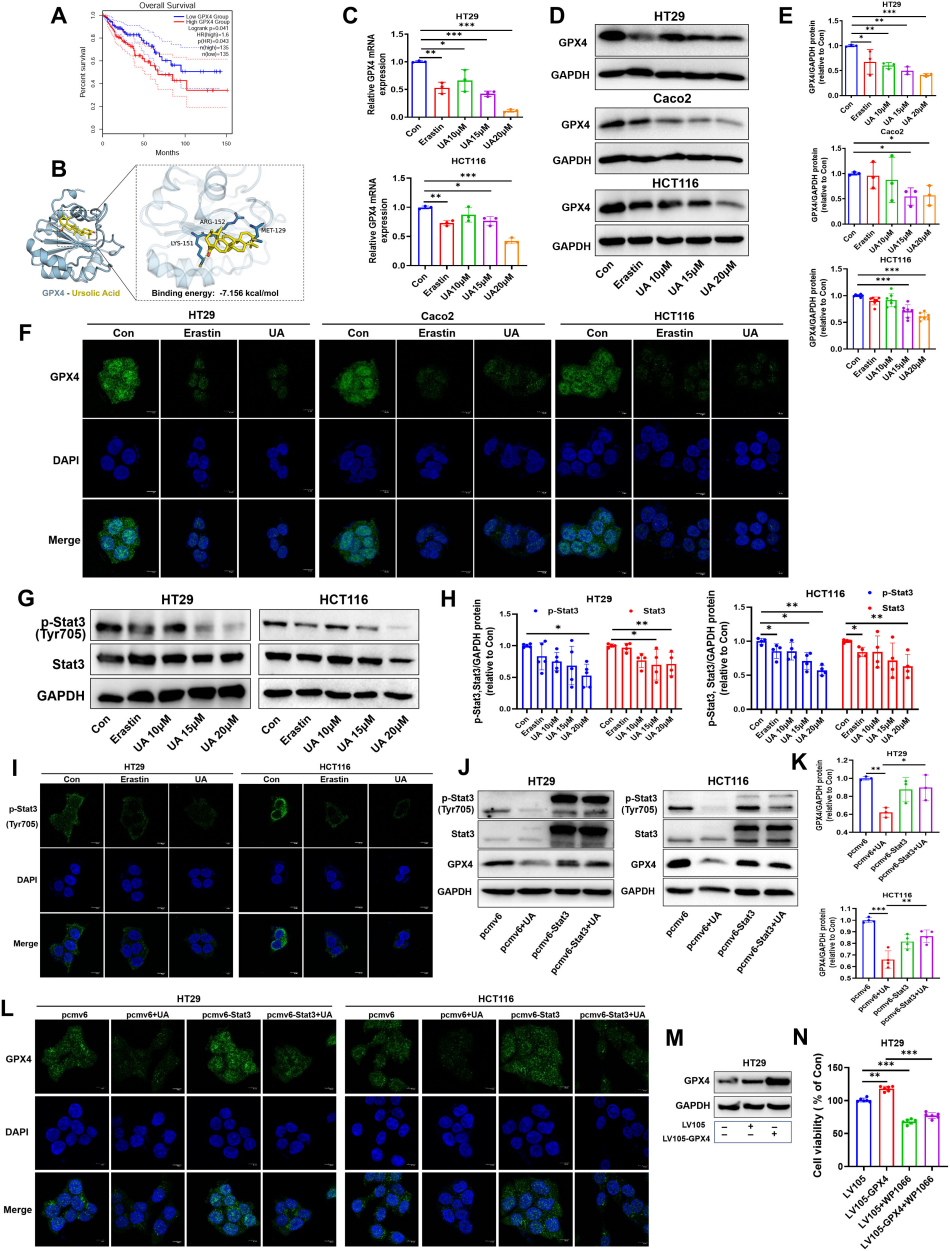
UA inhibits Stat3/GPX4 regulatory axis. **(A)** Database analysis of overall survival of COAD patients of low GPX4 group and high GPX4 group. **(B)** Molecular docking analysis of GPX4 and UA. Binding energy is -7.156 kcal/mol. **(C)** GPX4 mRNA levels was tested via qRT-PCR (n=3). **(D, E)** GPX4 protein expression levels were detected by Western blot (n=3). **(F)** GPX4 protein expression displayed by immunofluorescence staining assay. **(G, H)** Protein expression levels of p-Stat3 and Stat3 were detected by Western blot (n=4). **(I)** Protein expression of p-Stat3 were detected by immunofluorescence. **(J, K)** Cells were transfected with pcmv6-Stat3 plasmids or empty plasmids for 24 h, then treated with UA for another 24 h. Then, GPX4 protein expression levels were detected by Western blot (n=3). **(L)** GPX4 protein expression conditions were displayed by immunofluorescence assay after plasmids transfection and UA intervention. **(M, N)** Cells were transfected with LV105-GPX4 plasmids or empty plasmids for 24 h, then treated with WP1066 for another 24 h. Then, cell viability was calculated (n=6). *p < 0.05, **p < 0.01, ***p < 0.001.

Glutathione peroxidase 4 (GPX4), a vital repressor of ferroptosis. The availability of GSH closely mediates GPX4 activity (21). GPX4 plays crucial role in promoting cancer cell survival, especially in cancer phenotypes with feature of dedifferentiated states or stem cell-like (22). In line with this, COAD patients with higher GPX4 mRNA levels were correlated with lower OS (**Figure 4A**). Interestingly, molecular docking result showed that there was quite high binding affinity between GPX4 and UA. The binding energy is -7.156 kcal/mol. (**Figure 4B**). Since above results indicated that UA could downregulate GSH levels and may bind to GPX4, so we wondered whether UA could influence GPX4 expression in CRC. The results suggested that UA can evidently reduce GPX4 mRNA levels and protein levels, closely related to dose (**Figures 4C–E**). Images generated by immunofluorescence staining assay also demonstrated that UA was able to inhibit cellular GPX4 protein expression (**Figure 4F**).

Next, we were curious about the upstream molecules that regulate GPX4 expression. Firstly, we focused on Stat3. It is confirmed that Stat3 can bind GPX4 promoter region, then regulate GPX4 protein expression in oropharyngeal cancer cell by chromatin immunoprecipitation assay (23). In present research, immunoblotting showed that the expressions of p-Stat3, Stat3 in HT29 and HCT116 cells decreased significantly in response to UA treatment (**Figures 4G, H**). Cellular immunofluorescence images also suggested that UA treatment visibly attenuated p-Stat3 expression levels (**Figure 4I**). Stat3 overexpression can counteract UA-induced inhibition of GPX4 protein expression, as shown by immunoblotting and immunofluorescence experiment (**Figures 4J–L**). Subsequently, we validated the effects of GPX4 overexpression and Stat3 inhibitor WP1066 on CRC cells growth. The corresponding results showed that overexpression of GPX4 promoted CRC cells growth, while Stat3 inhibitor inhibited cells growth (**Figures 4M, N**). Based on above results, we figured out that UA can be regarded as an inhibitory factor in CRC proliferation and exert function by suppressing the Stat3/GPX4 axis.

### UA promotes miR-214-3p expression levels in CRC cells

**Figure 5:**
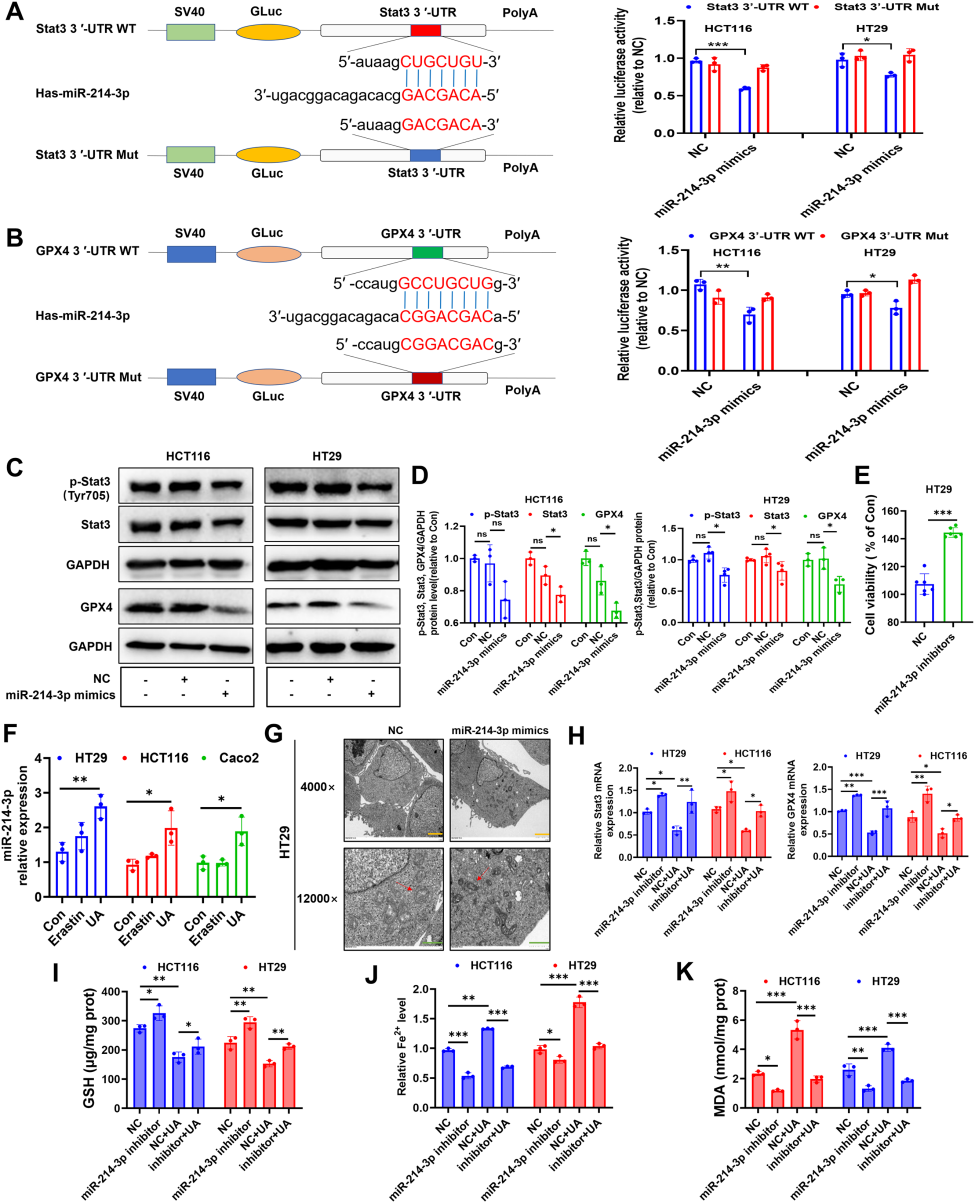
UA increases miR-214-3p expression in CRC cells. **(A)** The luciferase reporter constructed with mutant or wild type Stat3 3’UTR sequences were shown. Cells were transfected with Stat3 3’UTR-WT or Stat3 3’UTR-Mut vectors for 24 h, then transfected with miR-214-3p mimics or NC for another 24 h. The luciferase activity was detected (n=3). **(B)** Cells were transfected with GPX4 3’UTR-WT or GPX4 3’UTR-Mut vectors for 24 h, then transfected with miR-214-3p mimics or NC for another 24 h. The luciferase activity was detected (n=3). **(C, D)** Cells were transfected with miR-214-3p mimics or NC for 24 h, then protein expression levels of p-Stat3, Stat3 and GPX4 were detected by Western blot (n=3). **(E)** Cells were transfected with miR-214-3p inhibitors or NC for 48 h, then cell viability was measured (n=6). **(F)** MiR-214-3p levels were tested via qRT-PCR (n=3). **(G)** TEM observation of mitochondria in HT29 cells transfected with miR-214-3p mimics or NC. (Magnification=×4.0k [scale bar, 2 μm] and 12.0k [scale bar, 1 μm]). **(H)** Cells were transfected with miR-214-3p inhibitors or NC for 24 h, then exposed to UA treatment for another 24 h. Stat3 mRNA and GPX4 mRNA levels were tested via qRT-PCR (n=3). Cellular GSH levels **(I)**, Fe^2+^ levels **(J)** and MDA levels **(K)** were detected after miR-214-3p inhibitor or NC transfection with or without UA treatment (n=3). *p < 0.05, **p < 0.01, ***p < 0.001. ns, not significant.

It is widely accepted that microRNA (miRNA) can target specific mRNAs, then interfere their translation. We searched on miRmap website (https://mirmap.ezlab.org/) and found that miR-214-3p can target GPX4 (miRmap score: 93.46) and Stat3 (miRmap score: 45.89), with strong and moderate repression strength, respectively. Results from dual-luciferase gene assay inferred that miR-214-3p can act as sponge of GPX4 mRNA and Stat3 mRNA (**Figures 5A, B**). Next, we discovered decreased levels of p-Stat3, Stat3 and GPX4 under miR-214-3p mimics administration (**Figures 5C, D**). Transfection of this gene inhibitors into HT29 cells resulted in accelerating cell proliferation (**Figure 5E**).

Surprisingly, UA can significantly increase miR-214-3p expression levels in all three CRC cells (**Figure 5F**). In addition, results from TEM images showed that mitochondrial volume decreased, bilayer membrane density increased, mitochondrial cristae decreased after intervention with miR-214-3p mimics. (**Figure 5G**). To fully validate miR-214-3p’s role in ferroptosis, we performed a series of inhibitor studies and rescue experiments. Downregulation of miR-214-3p can reverse UA-reduced Stat3 mRNA and GPX4 mRNA expression levels (**Figure 5H**). Subsequently, we figured out that miR-214-3p inhibitors can upregulate Stat3 and GPX4 protein expression levels (**Supplementary Figures S1A, B**). GSH levels, Fe^2+^ levels and MDA levels were also counteracted with addition of miR-214-3p inhibitors, compared with UA alone treatment group (**Figures 5I–K**).

### UA inhibits HT29 cells xenograft tumor growth

**Figure 6:**
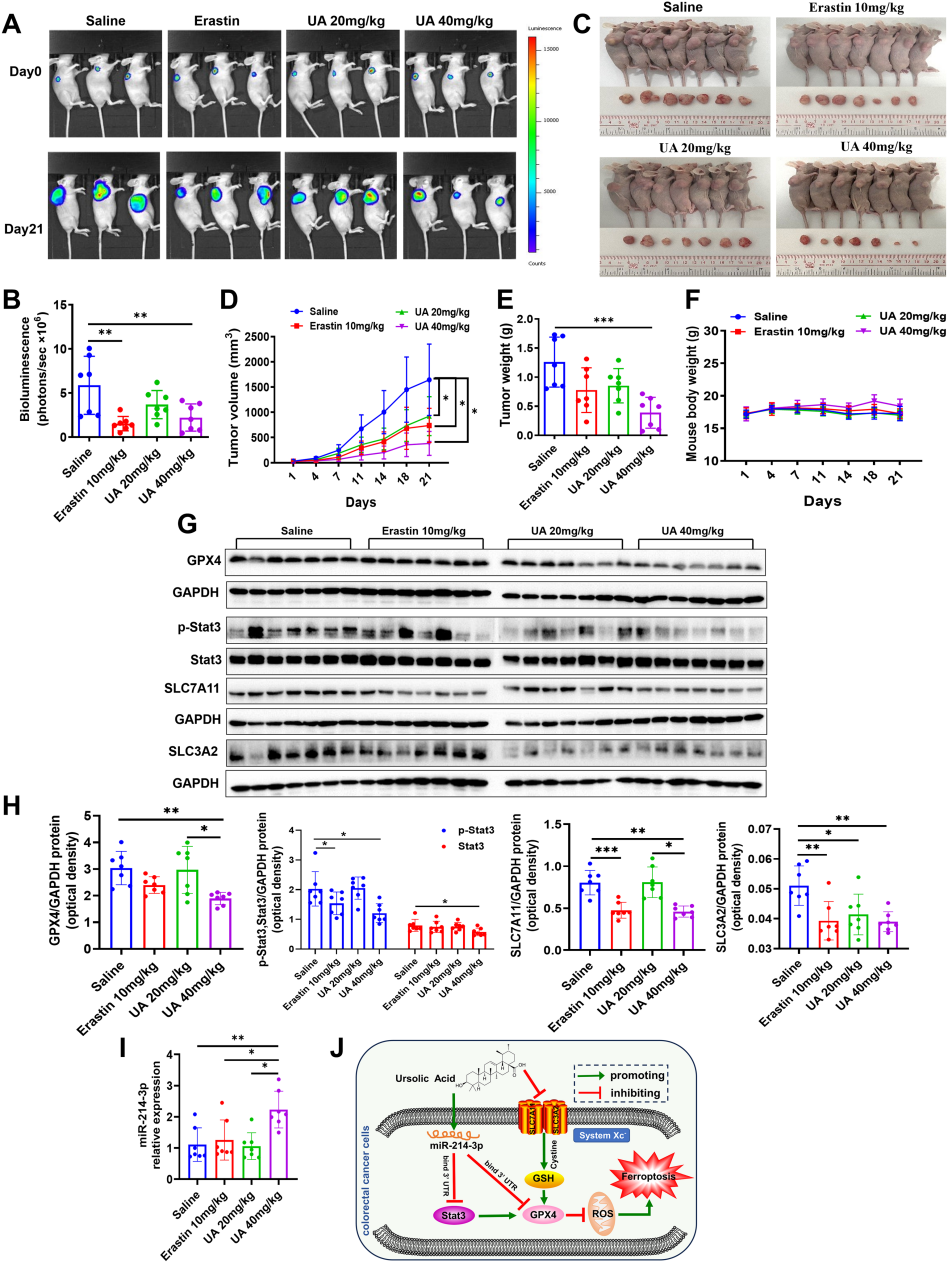
UA can suppress HT29-luc cells xenograft model tumor growth. **(A, B)** Tumor growth status was measured by detection of HT29-luc cells bioluminescence signals. **(C)** Tumor tissues were harvested and photographed after 21 days intragastric administration. **(D)** Tumor volume. **(E)** Tumor weight. **(F)** Mice body weight was recorded every three days. **(G, H)** Protein expression levels of GPX4, p-Stat3, Stat3, SLC7A11 and SLC3A2 in tumor tissues were detected by Western blot. **(I)** MiR-214-3p levels in tumor tissues were tested by qRT-PCR. **(J)** The diagram showed that UA inhibited CRC cells growth and induced ferroptosis through suppressing SLC7A11, SLC3A2, GPX4, Stat3 expression, but increasing miR-214-3p expression. There is regulatory axis among these targets. n=7. *p < 0.05, **p < 0.01, ***p < 0.001.

We further verified the effect of UA on CRC tumor in mice xenograft model. The HT29-luc cells were seeded to the right flank of mice. Of note, the vehicle used for UA delivery in animal experiments was saline. According to the results, it was found that compared to the control, mice from UA (40 mg/kg) treatment group showed weaker luciferase activity, smaller tumor volume, and weight. (**Figures 6A–E**). In addition, there exists no obvious difference in body weight among the groups (**Figure 6F**). In accordance with cell experiment results, lower protein levels of GPX4, p-Stat3, Stat3, SLC7A11 and SLC3A2 were found in nude mice tumor tissues from UA (40 mg/kg) group (**Figures 6G, H**). Moreover, miR-214-3p expression levels increased obviously in UA higher dose group (**Figure 6I**). In summary, it can be concluded that UA may repress CRC cells proliferation and induce ferroptosis via regulation of system xc^-^ and miR-214-3p/Stat3/GPX4 axis (**Figure 6J**).

## Discussion

Colorectal cancer (CRC) represents a prevalent form of malignant neoplasm globally, posing a substantial threat to human health. Thus, the pursuit of more effective and safer therapeutic strategies is imperative (24). UA, as an active compound extracted from HDW, has already shown the ability to interfere cancer cells malignancy in many reports (25). A recent investigation revealed that combined treatment with UA and Solasodine effectively suppressed the AKT1/ERK1/2-GSK-3β-β-catenin signaling pathway, thereby promoting apoptosis, autophagy, and exhibiting anti-metastatic properties through the inhibition of AKT1 and ERK1/2 (26).A study demonstrated that UA suppressed the proliferation of colorectal cancer (CRC) cells, affected the cell cycle, and enhanced apoptosis through the modulation of the miR-140-5p/TGF-β3 pathway. This effect may be associated with the suppression of the Wnt/β-catenin signaling pathway (27). Additionally, another investigation highlighted that UA can impede the metastasis of colon cancer by enhancing the ubiquitination-mediated degradation of ADP-ribosylation factor-like GTPase 4C (ARL4C) (28). In our research, we demonstrated that UA effectively inhibited CRC proliferation and induced both ferroptosis and apoptosis. We employed the apoptosis inhibitor Z-VAD-FMK and Fer-1 to perform rescue assays. Our findings indicated that only Fer-1 was able to partially mitigate the suppressive impact of UA on the viability of colorectal cancer (CRC) cells, thereby suggesting that ferroptosis serves as the primary mechanism involved. Ferroptosis is recognized as a novel form of regulated cell death (RCD) characterized by iron-dependent accumulation of lipid peroxides localized to cellular membranes (29). Accumulating evidence have gradually validated ferroptosis as an effective target for tumor therapy. We observed higher levels of ROS, Fe^2+^ iron and MDA, and downregulated levels of system xc^-^ and GPX4 after UA therapy, which altogether indicated occurrence of lipid peroxidation.

To investigate the mechanisms underlying ferroptosis induced by UA, our research concentrated on the pertinent regulatory axis. Previous studies have established that Stat3 interacts with the promoters of GPX4, SLC7A11, and FTH1, thereby modulating their expression levels in gastric cancer, which leads to the formation of a Stat3-ferroptosis regulatory axis (30). Results from another study demonstrates that thiostrepton induces ferroptosis via Stat3/GPX4 axis in pancreatic cancer cells (31). We found UA can reduce p-Stat3, Stat3 expression levels and overexpression of Stat3 counteracted UA-inhibited GPX4 expression. MiR-214-3p is a prognostic biomarker in many kinds cancers, such as lung cancer (32), cervical cancer (33), bladder cancer (34). Nevertheless, the function of miR-214-3p played in CRC remains unknown. In the current study, we observed a significant increase in miR-214-3p levels following UA treatment. Furthermore, the application of miR-214-3p mimics was found to affect the protein expression of Stat3 and GPX4. Bioinformatics predictions and results from luciferase reporter assays indicated that miR-214-3p can bind to the 3’ untranslated region (3’UTR) of Stat3 and GPX4 mRNA. Nonetheless, the specific targeting sites of Stat3 on the GPX4 promoter, along with its potential mediation of system xc^-^ or other proteins, merit further investigation. We recognize that there may be potential confounding factors or alternative interpretations of our findings, including the impact of UA on additional pathways that were not addressed in this study.

Nowadays, UA’s clinical application is hindered by inadequate permeability and suboptimal oral bioavailability. Innovative nano-formulations, including polymeric micelles and liposomes, have emerged as viable strategies to enhance the delivery of UA to tumor sites while simultaneously improving the compound’s stability (35). Numerous nano-formulations have been created to bolster stability and augment the effectiveness of drug release. By reducing the accumulation of drugs at non-target sites, the clinical efficacy of UA is significantly enhanced. Liposomes have the potential to mimic cellular membranes, thereby facilitating the drug-delivery mechanism. Polymer micelles encapsulating UA were synthesized utilizing mPEG-PLA (methoxy polyethylene glycol poly lactic acid) with the aim of specifically targeting hepatocellular carcinoma cells. This novel delivery system exhibited exceptional stability and maintained a uniform spherical morphology, facilitating a controlled release profile at varying pH values (7.5 and 5.5), which contributed to an enhanced accumulation within tumor tissues. Notably, this formulation effectively suppressed the proliferation of HepG2 cells while sparing normal hepatic cells from cytotoxicity (36). Preliminary Phase I clinical trials evaluating UA nano-formulations have revealed acceptable toxicity profiles and linear pharmacokinetics, suggesting both safety and potential efficacy (37, 38). For example, a research study examined the safety profile and antitumor efficacy of multiple doses of UA delivered via liposomes (UAL) in patients with advanced solid tumors. Participants received intravenous infusions of UAL for a consecutive period of 14 days within a 21-day treatment cycle. To evaluate the effectiveness and tolerability of the multiple dosing regimen, twenty-one patients were allocated into one of three sequential dosage cohorts (56, 74, and 98 mg/m^2^). Furthermore, an additional cohort of eight individuals was assessed for the pharmacokinetics of UAL at the 74 mg/m^2^ dosage. The findings from the pharmacokinetic assessment revealed no evidence of UAL accumulation within the body. Consequently, UAL was recognized as a well-tolerated therapeutic agent with manageable toxicity, which may enhance the remission rates among patients (39). In future, investigations should focus on the refinement of UA nano-formulations to maximize their clinical applicability. Furthermore, integrating UA with other therapeutic approaches, such as immunotherapy and targeted therapies, may yield novel treatment paradigms for CRC cancer.

Notably, this study has some limitations. We acknowledge the limitation regarding the generalizability of our findings due to the testing of only three CRC cell lines and small sample size in animal experiments. In subsequent studies, we will explore a broader range of cell lines and larger sample size. Moreover, we plan to incorporate immune-competent models to better understand tumor-immune interactions relevant to ferroptosis.

## Conclusion

In total, this study investigated the tumor-blocking effect of UA, a component of traditional Chinese medicine *Hedyotis diffusa* Willd, discovered the involvement of CRC cellular ferroptosis. Analysis on possible mechanisms verified the potential adjust axis of miR-214-3p/Stat3/GPX4 and inhibition of system xc^-^ in UA-stimulated ferroptosis. The obtained results may come up with a novel therapeutic method for colorectal cancer.

## Data Availability

The raw sequences data reported in this research was deposited in public repository NCBI SRA with accession number PRJNA1344781.

## References

[1] Bray F Laversanne M Sung H Ferlay J Siegel RL Soerjomataram I . Global cancer statistics 2022: GLOBOCAN estimates of incidence and mortality worldwide for 36 cancers in 185 countries. CA: Cancer J Clin. (2024) 74:229–63. doi: 10.3322/caac.21834, PMID: 38572751

[2] Biller LH Schrag D . Diagnosis and treatment of metastatic colorectal cancer: A review. Jama. (2021) 325:669–85. doi: 10.1001/jama.2021.0106, PMID: 33591350

[3] Dekker E Tanis PJ Vleugels JLA Kasi PM Wallace MB . Colorectal cancer. Lancet (London England). (2019) 394:1467–80. doi: 10.1016/S0140-6736(19)32319-0, PMID: 31631858

[4] Song Y Wang H Pan Y Liu T . Investigating the multi-target pharmacological mechanism of hedyotis diffusa willd acting on prostate cancer: A network pharmacology approach. Biomolecules. (2019) 9:591. doi: 10.3390/biom9100591, PMID: 31600936 PMC6843553

[5] Meng QX Roubin RH Hanrahan JR . Ethnopharmacological and bioactivity guided investigation of five TCM anticancer herbs. J Ethnopharmacol. (2013) 148:229–38. doi: 10.1016/j.jep.2013.04.014, PMID: 23623820

[6] Lee HZ Bau DT Kuo CL Tsai RY Chen YC Chang YH . Clarification of the phenotypic characteristics and anti-tumor activity of Hedyotis diffusa. Am J Chin Med. (2011) 39:201–13. doi: 10.1142/S0192415X11008750, PMID: 21213409

[7] Sandhu SS Rouz SK Kumar S Swamy N Deshmukh L Hussain A . Ursolic acid: a pentacyclic triterpenoid that exhibits anticancer therapeutic potential by modulating multiple oncogenic targets. Biotechnol Genet Eng Rev. (2023) 39:729–59. doi: 10.1080/02648725.2022.2162257, PMID: 36600517

[8] Lin JH Chen SY Lu CC Lin JA Yen GC . Ursolic acid promotes apoptosis, autophagy, and chemosensitivity in gemcitabine-resistant human pancreatic cancer cells. Phytotherapy research: PTR. (2020) 34:2053–66. doi: 10.1002/ptr.6669, PMID: 32185829

[9] Kim GH Kan SY Kang H Lee S Ko HM Kim JH . Ursolic acid suppresses cholesterol biosynthesis and exerts anti-cancer effects in hepatocellular carcinoma cells. Int J Mol Sci. (2019) 20:4767. doi: 10.3390/ijms20194767, PMID: 31561416 PMC6802365

[10] Liao WL Liu YF Ying TH Shieh JC Hung YT Lee HJ . Inhibitory effects of ursolic acid on the stemness and progression of human breast cancer cells by modulating argonaute-2. Int J Mol Sci. (2022) 24:366. doi: 10.3390/ijms24010366, PMID: 36613808 PMC9820512

[11] Schwabe RF Luedde T . Apoptosis and necroptosis in the liver: a matter of life and death. Nat Rev Gastroenterol Hepatol. (2018) 15:738–52. doi: 10.1038/s41575-018-0065-y, PMID: 30250076 PMC6490680

[12] Stockwell BR . Ferroptosis turns 10: Emerging mechanisms, physiological functions, and therapeutic applications. Cell. (2022) 185:2401–21. doi: 10.1016/j.cell.2022.06.003, PMID: 35803244 PMC9273022

[13] Yan H Talty R Johnson CH . Targeting ferroptosis to treat colorectal cancer. Trends Cell Biol. (2023) 33:185–8. doi: 10.1016/j.tcb.2022.11.003, PMID: 36473802

[14] Ma C Zhang X Mo X Yu Y Xiao Z Wu J . Xie-Bai-San increases NSCLC cells sensitivity to gefitinib by inhibiting Beclin-1 mediated autophagosome formation. Phytomedicine: Int J phytotherapy phytopharmacology. (2024) 125:155351. doi: 10.1016/j.phymed.2024.155351, PMID: 38232540

[15] Wu J Ma C Tang X Shi Y Liu Z Chai X . The regulation and interaction of PVT1 and miR181a-5p contributes to the repression of SP1 expression by the combination of XJD decoction and cisplatin in human lung cancer cells. BioMed Pharmacother. (2020) 121:109632. doi: 10.1016/j.biopha.2019.109632, PMID: 31707347

[16] Khatun J Gelles JD Chipuk JE . Dynamic death decisions: How mitochondrial dynamics shape cellular commitment to apoptosis and ferroptosis. Dev Cell. (2024) 59:2549–65. doi: 10.1016/j.devcel.2024.09.004, PMID: 39378840 PMC11469553

[17] Fang X Gao F Zheng L Xue FS Zhu T Zheng X . Reduced microRNA-744 expression in mast cell-derived exosomes triggers epithelial cell ferroptosis in acute respiratory distress syndrome. Redox Biol. (2024) 77:103387. doi: 10.1016/j.redox.2024.103387, PMID: 39378613 PMC11493202

[18] Zhou Q Meng Y Li D Yao L Le J Liu Y . Ferroptosis in cancer: From molecular mechanisms to therapeutic strategies. Signal transduction targeted Ther. (2024) 9:55. doi: 10.1038/s41392-024-01769-5, PMID: 38453898 PMC10920854

[19] Liu X Olszewski K Zhang Y Lim EW Shi J Zhang X . Cystine transporter regulation of pentose phosphate pathway dependency and disulfide stress exposes a targetable metabolic vulnerability in cancer. Nat Cell Biol. (2020) 22:476–86. doi: 10.1038/s41556-020-0496-x, PMID: 32231310 PMC7194135

[20] Parker JL Deme JC Kolokouris D Kuteyi G Biggin PC Lea SM . Molecular basis for redox control by the human cystine/glutamate antiporter system xc(). Nat Commun. (2021) 12:7147. doi: 10.1038/s41467-021-27414-1, PMID: 34880232 PMC8654953

[21] Ursini F Maiorino M . Lipid peroxidation and ferroptosis: The role of GSH and GPx4. Free Radical Biol Med. (2020) 152:175–85. doi: 10.1016/j.freeradbiomed.2020.02.027, PMID: 32165281

[22] Hangauer MJ Viswanathan VS Ryan MJ Bole D Eaton JK Matov A . Drug-tolerant persister cancer cells are vulnerable to GPX4 inhibition. Nature. (2017) 551:247–50. doi: 10.1038/nature24297, PMID: 29088702 PMC5933935

[23] Wu K Liu L Wu Z Huang Q Zhou L Xie R . Ascorbic acid induces ferroptosis *via* STAT3/GPX4 signaling in oropharyngeal cancer. Free Radical Res. (2024) 58:117–29. doi: 10.1080/10715762.2024.2320396, PMID: 38385781

[24] Doubeni CA Corley DA Jensen CD Levin TR Ghai NR Cannavale K . Fecal immunochemical test screening and risk of colorectal cancer death. JAMA network Open. (2024) 7:e2423671. doi: 10.1001/jamanetworkopen.2024.23671, PMID: 39028667 PMC11259903

[25] Alam M Ali S Ahmed S Elasbali AM Adnan M Islam A . Therapeutic potential of ursolic acid in cancer and diabetic neuropathy diseases. Int J Mol Sci. (2021) 22:12162. doi: 10.3390/ijms222212162, PMID: 34830043 PMC8621142

[26] Yang Y Liu P Jin Y Zhu H Wang M Jiang X . A combined treatment with Ursolic acid and Solasodine inhibits colorectal cancer progression through the AKT1/ERK1/2-GSK-3β-β-catenin axis. Phytomedicine. (2024) 135:156068. doi: 10.1016/j.phymed.2024.156068, PMID: 39515101

[27] Zhang T Xiang F Li X Chen Z Wang J Guo J . Mechanistic study on ursolic acid inhibiting the growth of colorectal cancer cells through the downregulation of TGF-β3 by miR-140-5p. J Biochem Mol Toxicol. (2024) 38:e23581. doi: 10.1002/jbt.23581, PMID: 38044485

[28] Zhang M Xiang F Sun Y Liu R Li Q Gu Q . Ursolic acid inhibits the metastasis of colon cancer by downregulating ARL4C expression. Oncol Rep. (2024) 51:27. doi: 10.3892/or.2023.8686, PMID: 38131251 PMC10777457

[29] Dixon SJ Lemberg KM Lamprecht MR Skouta R Zaitsev EM Gleason CE . Ferroptosis: an iron-dependent form of nonapoptotic cell death. Cell. (2012) 149:1060–72. doi: 10.1016/j.cell.2012.03.042, PMID: 22632970 PMC3367386

[30] Ouyang S Li H Lou L Huang Q Zhang Z Mo J . Inhibition of STAT3-ferroptosis negative regulatory axis suppresses tumor growth and alleviates chemoresistance in gastric cancer. Redox Biol. (2022) 52:102317. doi: 10.1016/j.redox.2022.102317, PMID: 35483272 PMC9108091

[31] Zhang W Gong M Zhang W Mo J Zhang S Zhu Z . Thiostrepton induces ferroptosis in pancreatic cancer cells through STAT3/GPX4 signalling. Cell Death Dis. (2022) 13:630. doi: 10.1038/s41419-022-05082-3, PMID: 35859150 PMC9300693

[32] Yang Y Li Z Yuan H Ji W Wang K Lu T . Reciprocal regulatory mechanism between miR-214-3p and FGFR1 in FGFR1-amplified lung cancer. Oncogenesis. (2019) 8:50. doi: 10.1038/s41389-019-0151-1, PMID: 31492847 PMC6731303

[33] Zhou Y Wang Y Lin M Wu D Zhao M . LncRNA HOTAIR promotes proliferation and inhibits apoptosis by sponging miR-214-3p in HPV16 positive cervical cancer cells. Cancer Cell Int. (2021) 21:400. doi: 10.1186/s12935-021-02103-7, PMID: 34320988 PMC8317292

[34] Cheng S Li C Liu L Liu X Li M Zhuo J . Dysregulation and antimetastatic function of circLRIG1 modulated by miR-214-3p/LRIG1 axis in bladder carcinoma. Biol direct. (2024) 19:20. doi: 10.1186/s13062-023-00446-x, PMID: 38454507 PMC10918934

[35] Chauhan A Pathak VM Yadav M Chauhan R Babu N Chowdhary M . Role of ursolic acid in preventing gastrointestinal cancer: recent trends and future perspectives. Front Pharmacol. (2024) 15:1405497. doi: 10.3389/fphar.2024.1405497, PMID: 39114347 PMC11303223

[36] Zhou M Yi Y Liu L Lin Y Li J Ruan J . Polymeric micelles loading with ursolic acid enhancing anti-tumor effect on hepatocellular carcinoma. J Cancer. (2019) 10:5820–31. doi: 10.7150/jca.30865, PMID: 31737119 PMC6843872

[37] Wang XH Zhou SY Qian ZZ Zhang HL Qiu LH Song Z . Evaluation of toxicity and single-dose pharmacokinetics of intravenous ursolic acid liposomes in healthy adult volunteers and patients with advanced solid tumors. Expert Opin Drug Metab Toxicol. (2013) 9:117–25. doi: 10.1517/17425255.2013.738667, PMID: 23134084

[38] Zhu Z Qian Z Yan Z Zhao C Wang H Ying G . A phase I pharmacokinetic study of ursolic acid nanoliposomes in healthy volunteers and patients with advanced solid tumors. Int J nanomedicine. (2013) 8:129–36. doi: 10.2147/IJN.S38271, PMID: 23319864 PMC3540956

[39] Qian Z Wang X Song Z Zhang H Zhou S Zhao J . A phase I trial to evaluate the multiple-dose safety and antitumor activity of ursolic acid liposomes in subjects with advanced solid tumors. BioMed Res Int. (2015) 2015:809714. doi: 10.1155/2015/809714, PMID: 25866811 PMC4383362

