# Supplementary figures and images for: Ursolic acid induces colorectal cancer cells ferroptosis via regulation of system xc- and miR-214-3p/Stat3/GPX4 axis
Source: Front Immunol. 2025 Nov 18;16:1674321. doi: 10.3389/fimmu.2025.1674321 (PMC12668971; doi:10.3389/fimmu.2025.1674321)

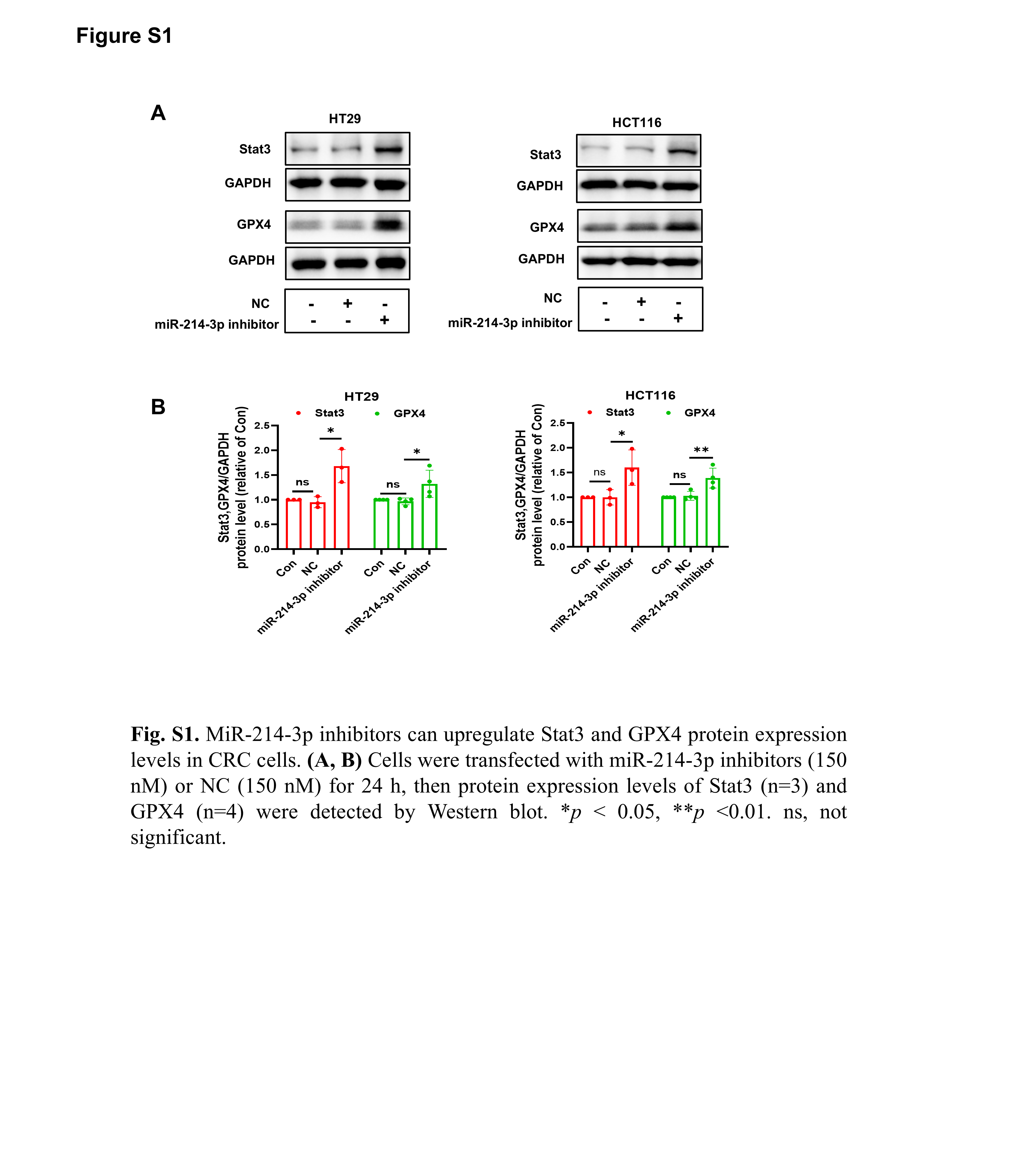

Supplement: Supplementary file 1 [file Image1.tif]

Fig 3B

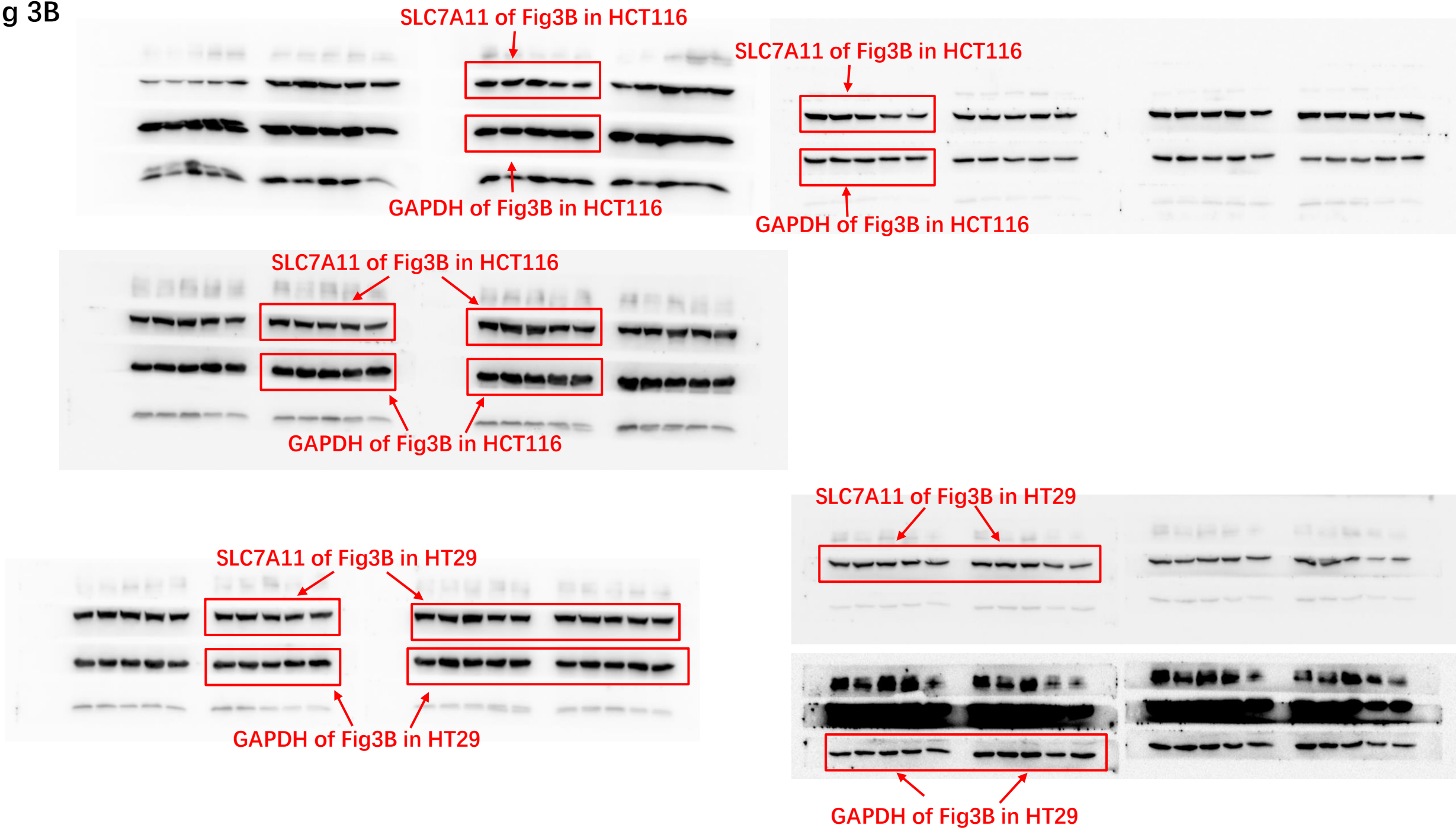

Fig 3F

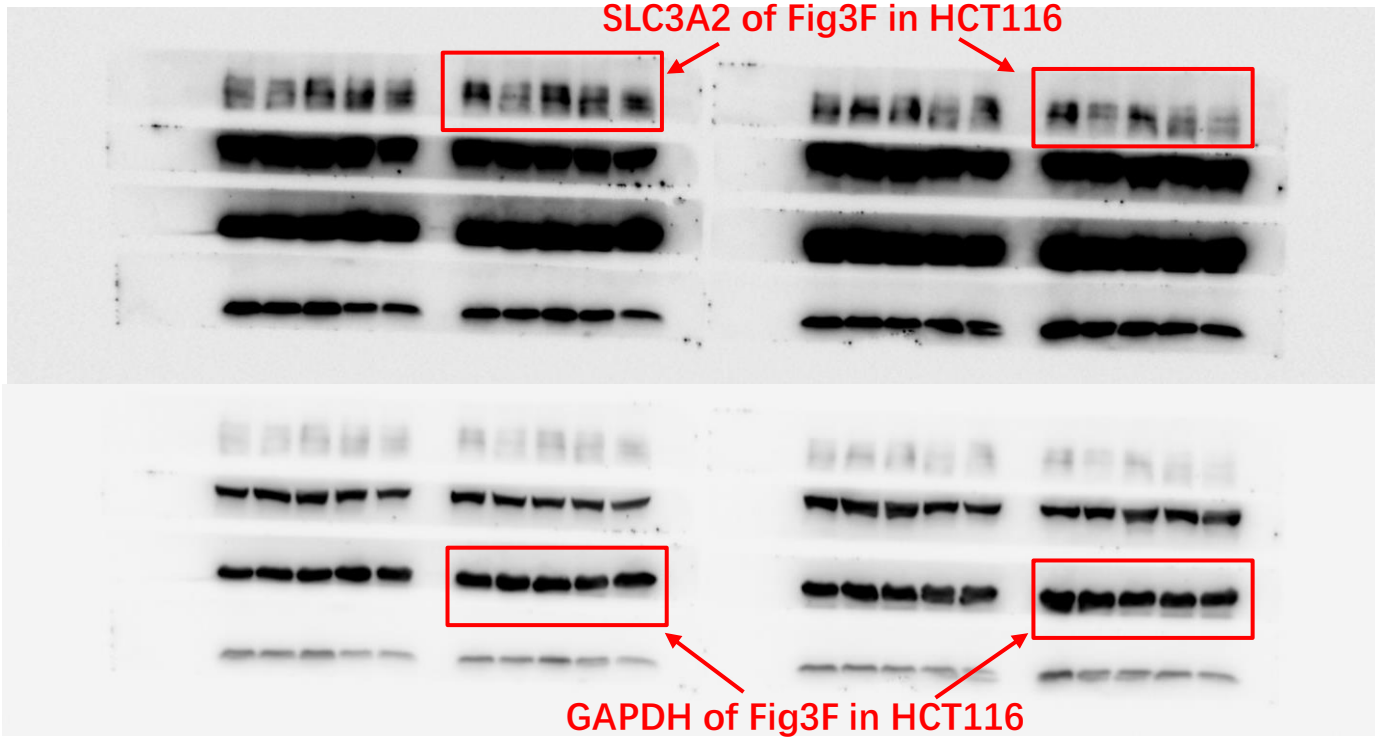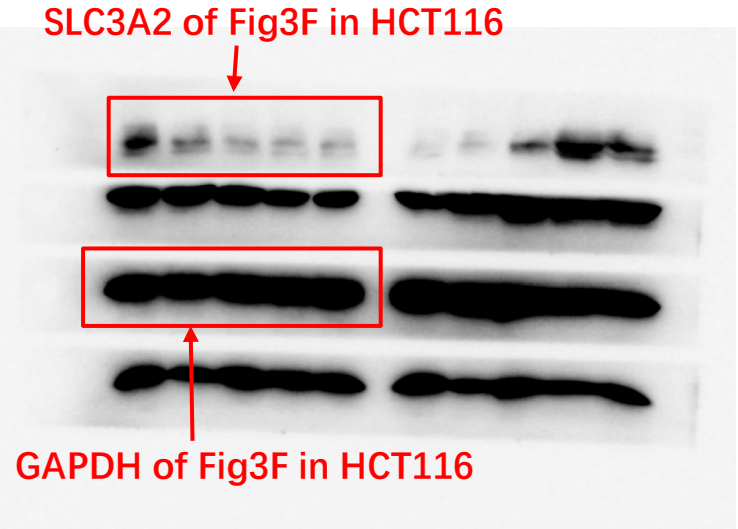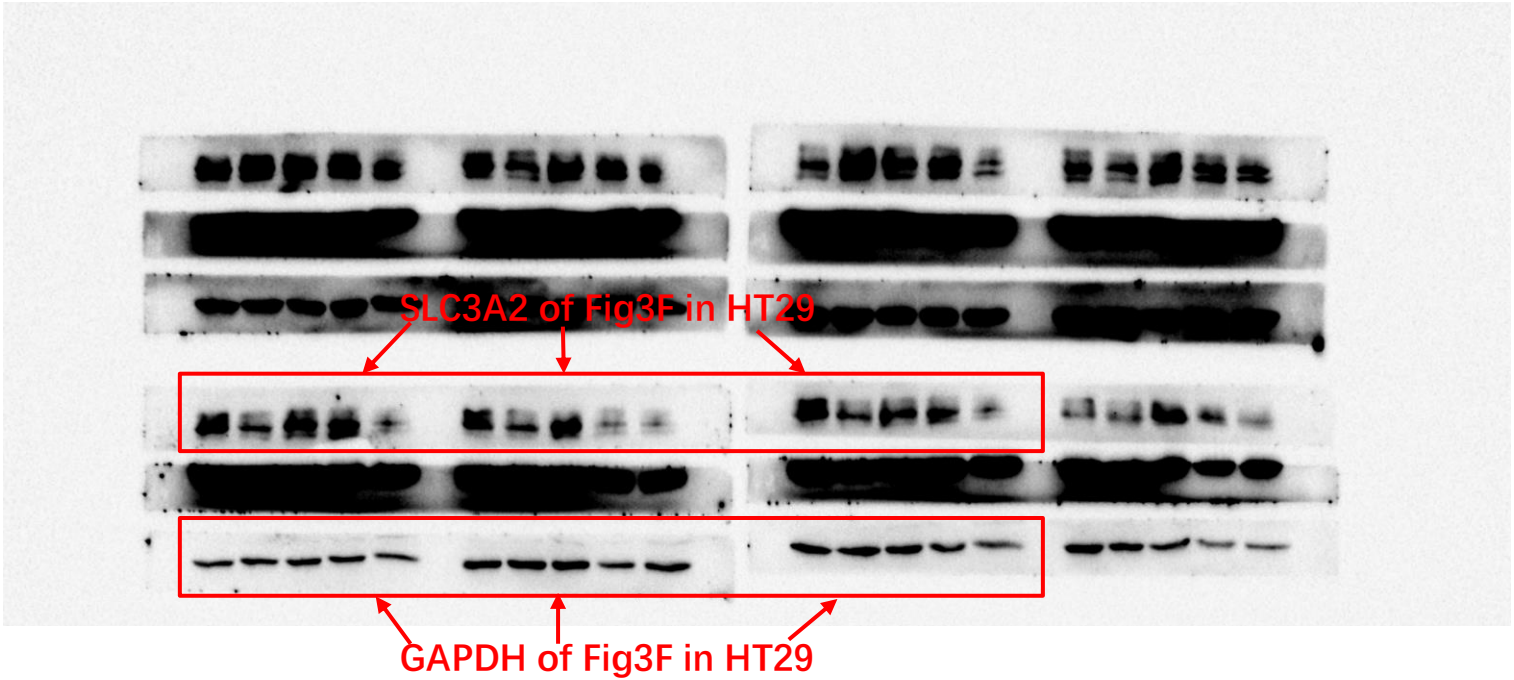

Fig 4D

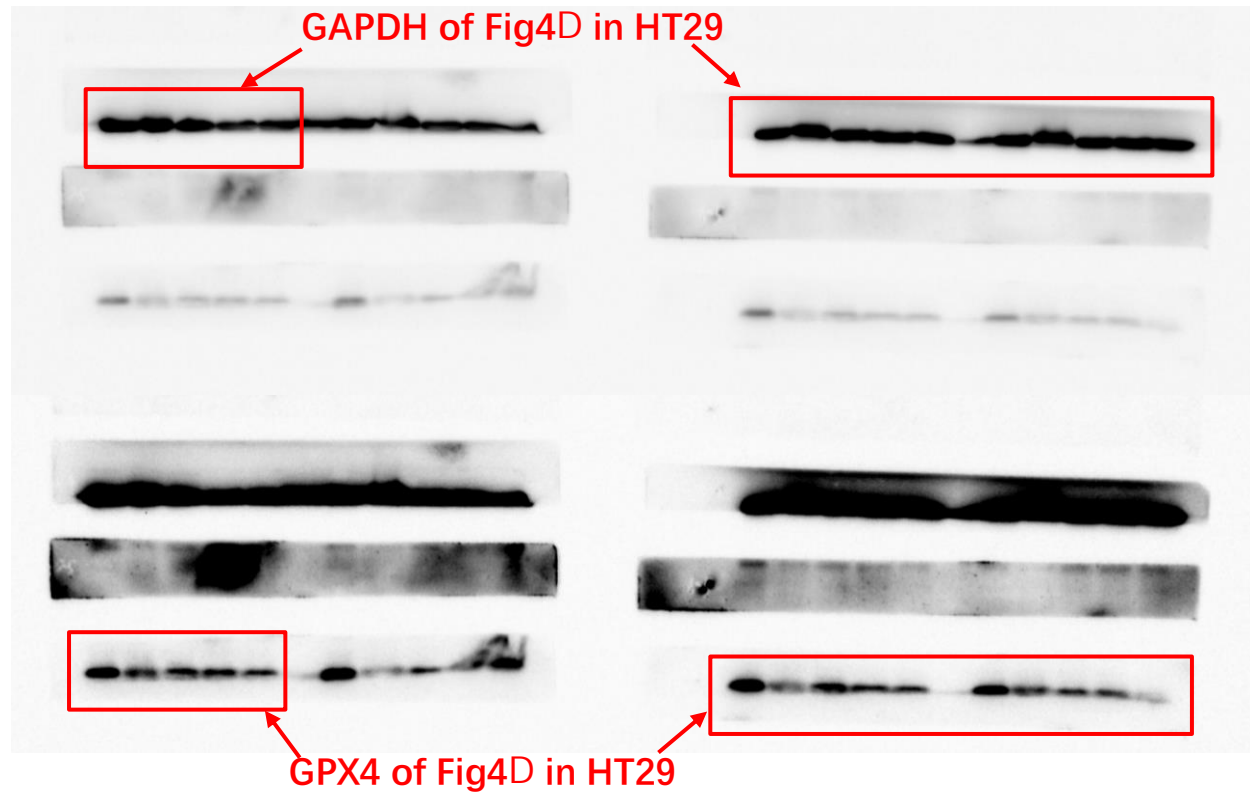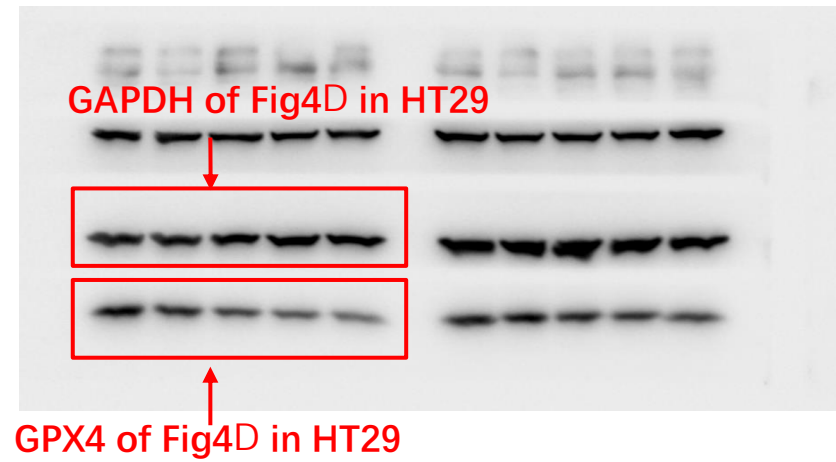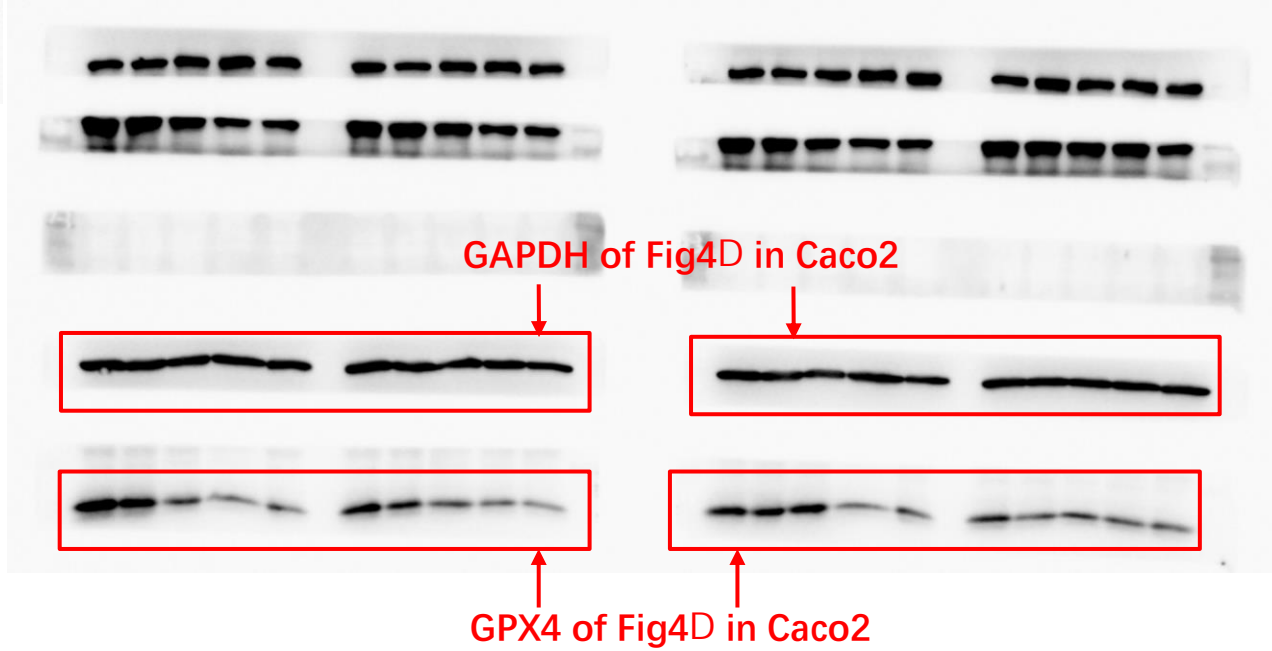

Fig 4D

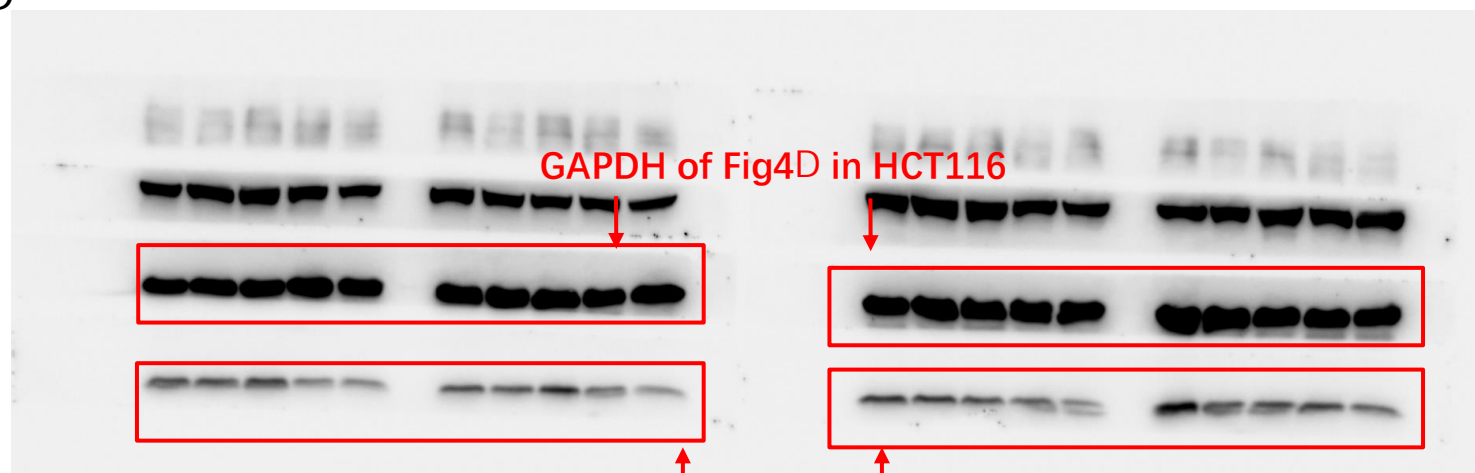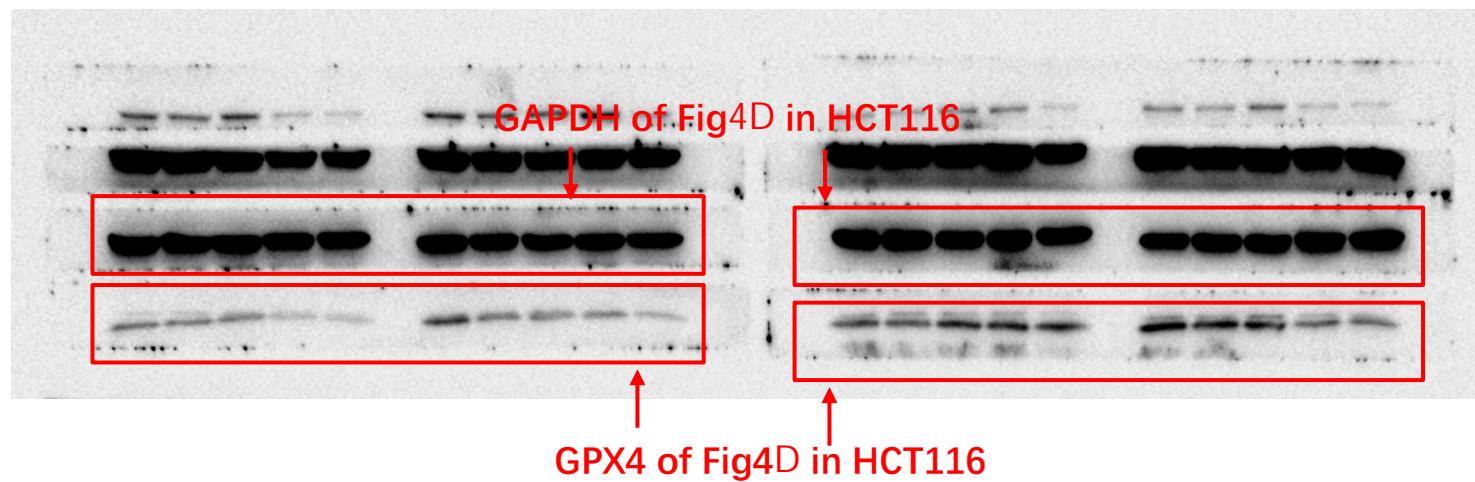

Fig 4G

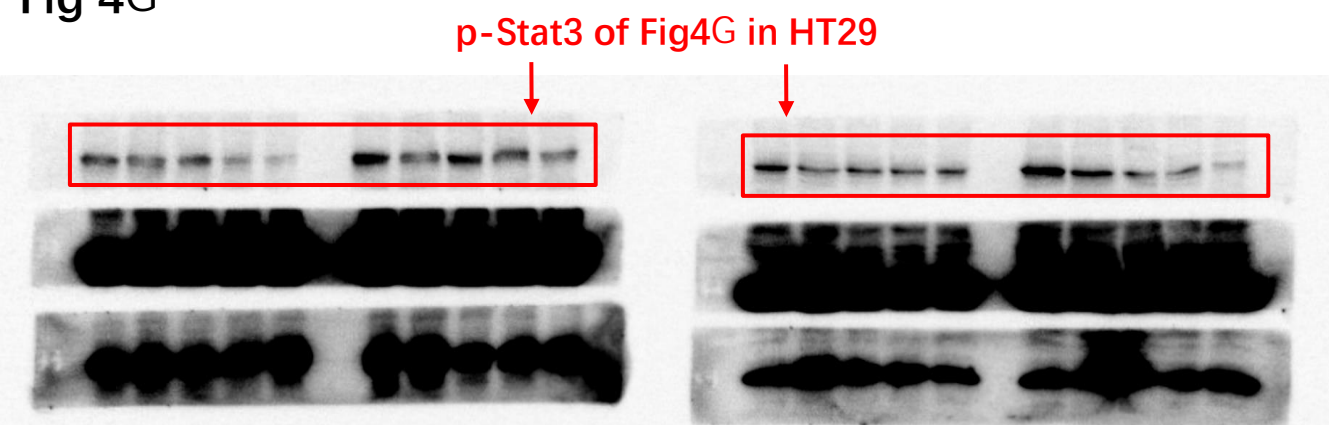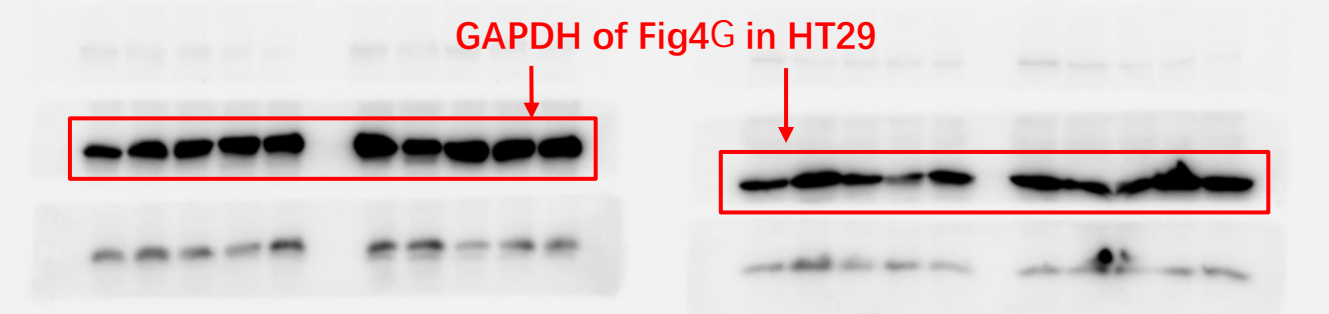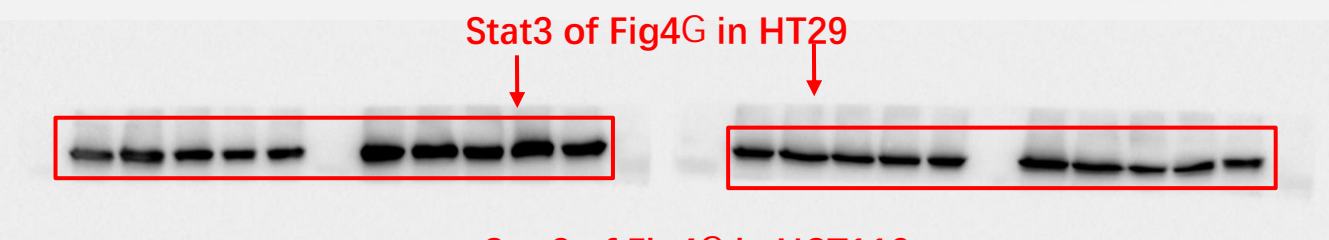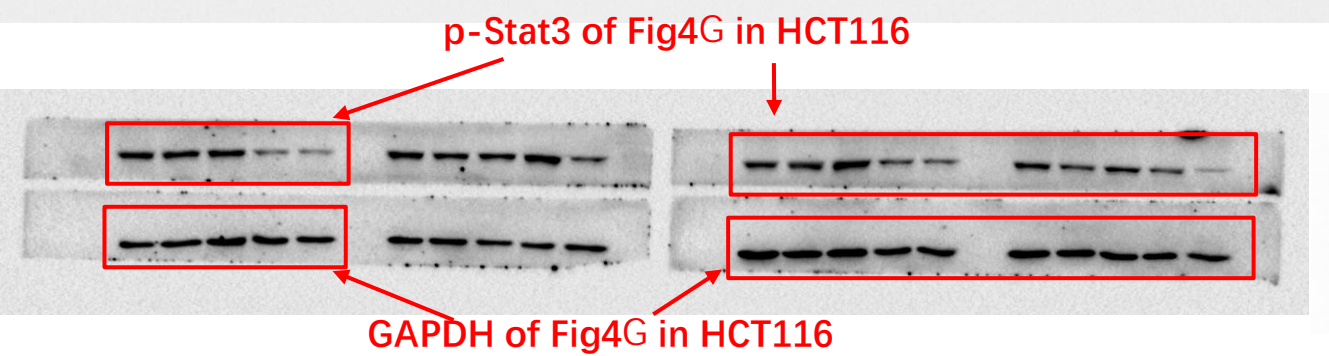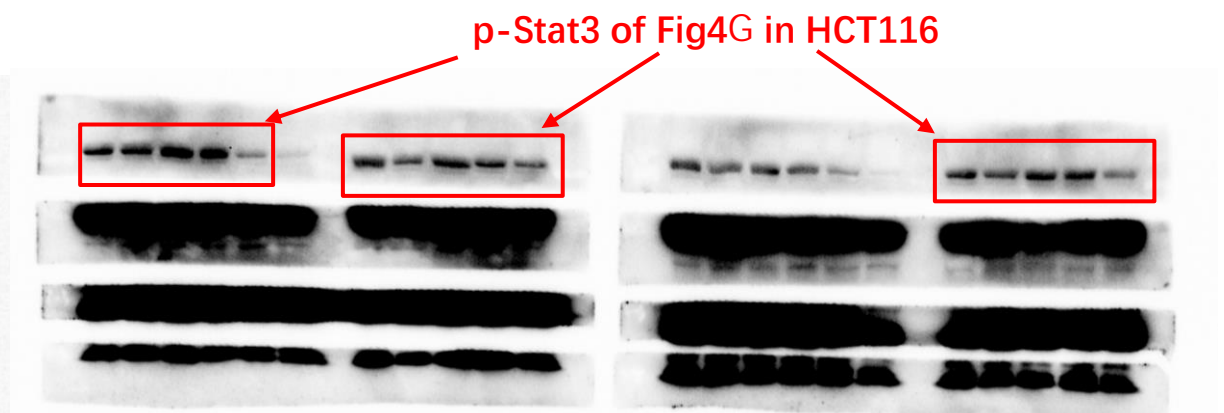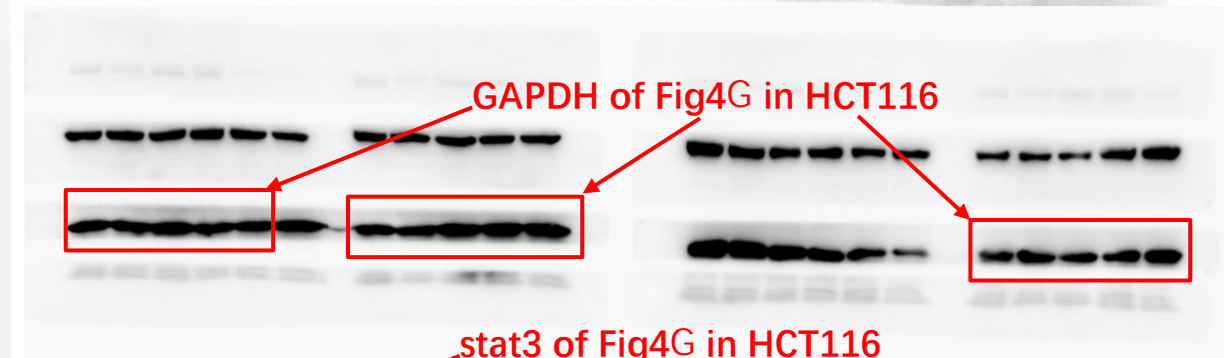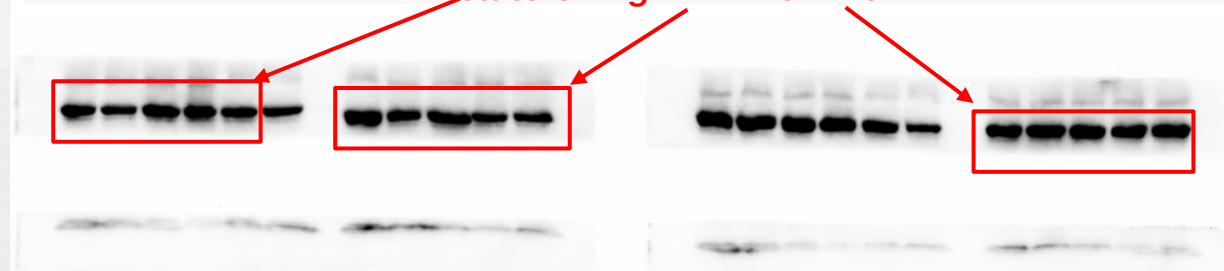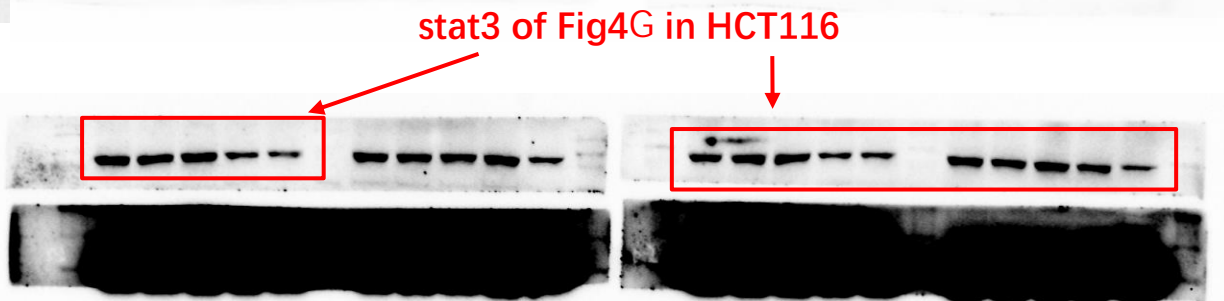

Fig 4J

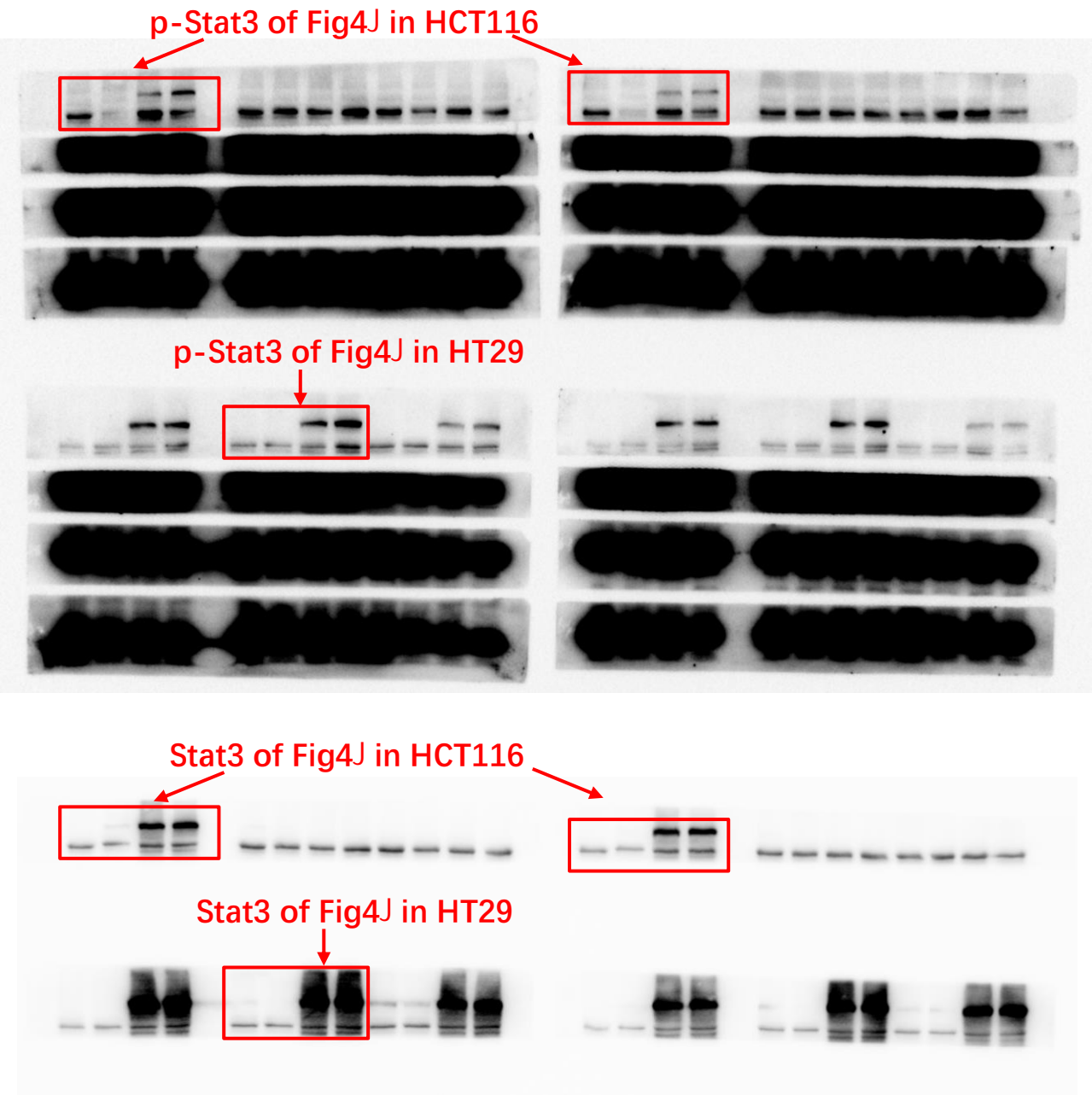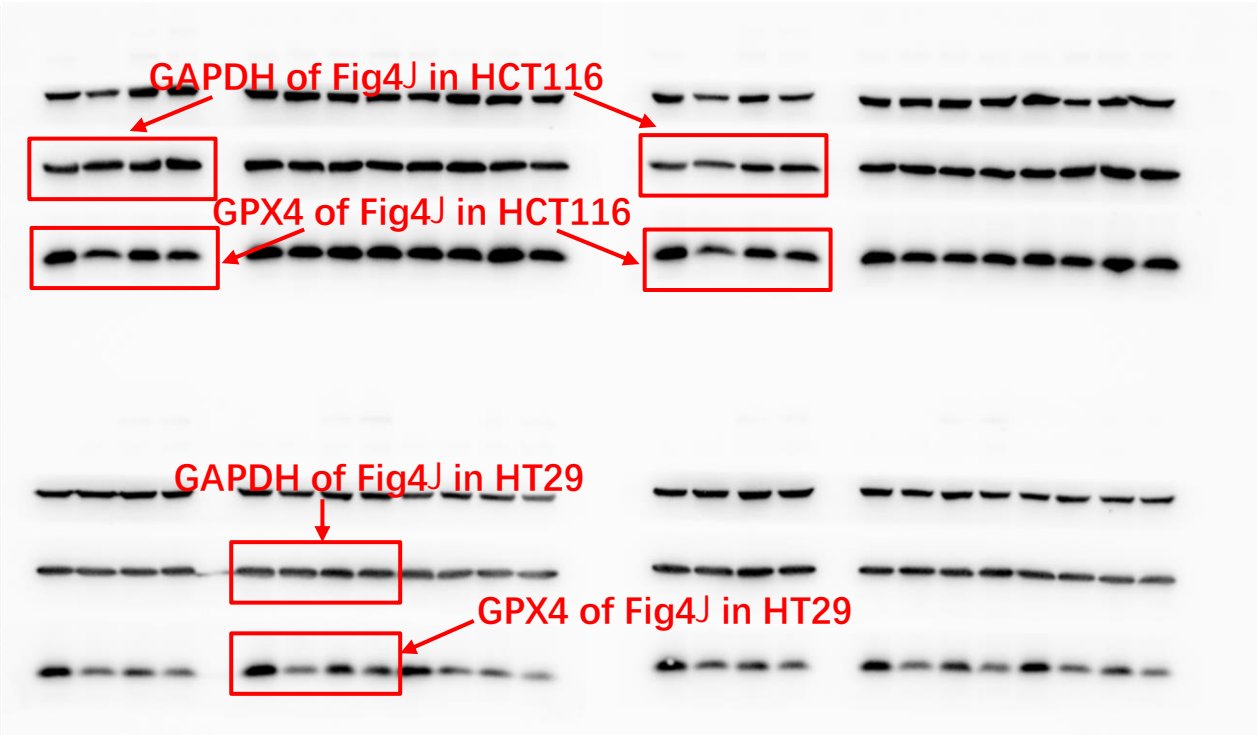

Fig 4J

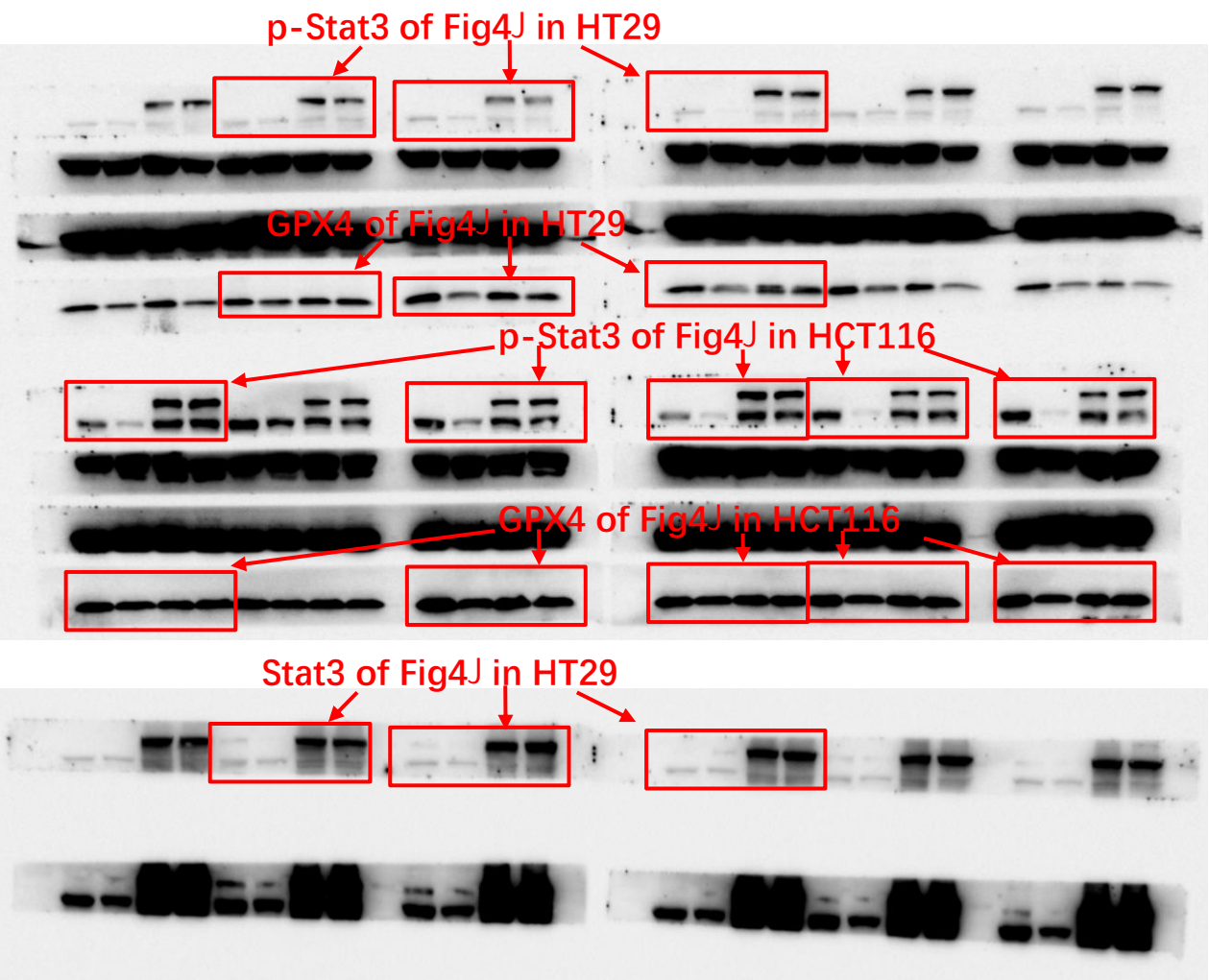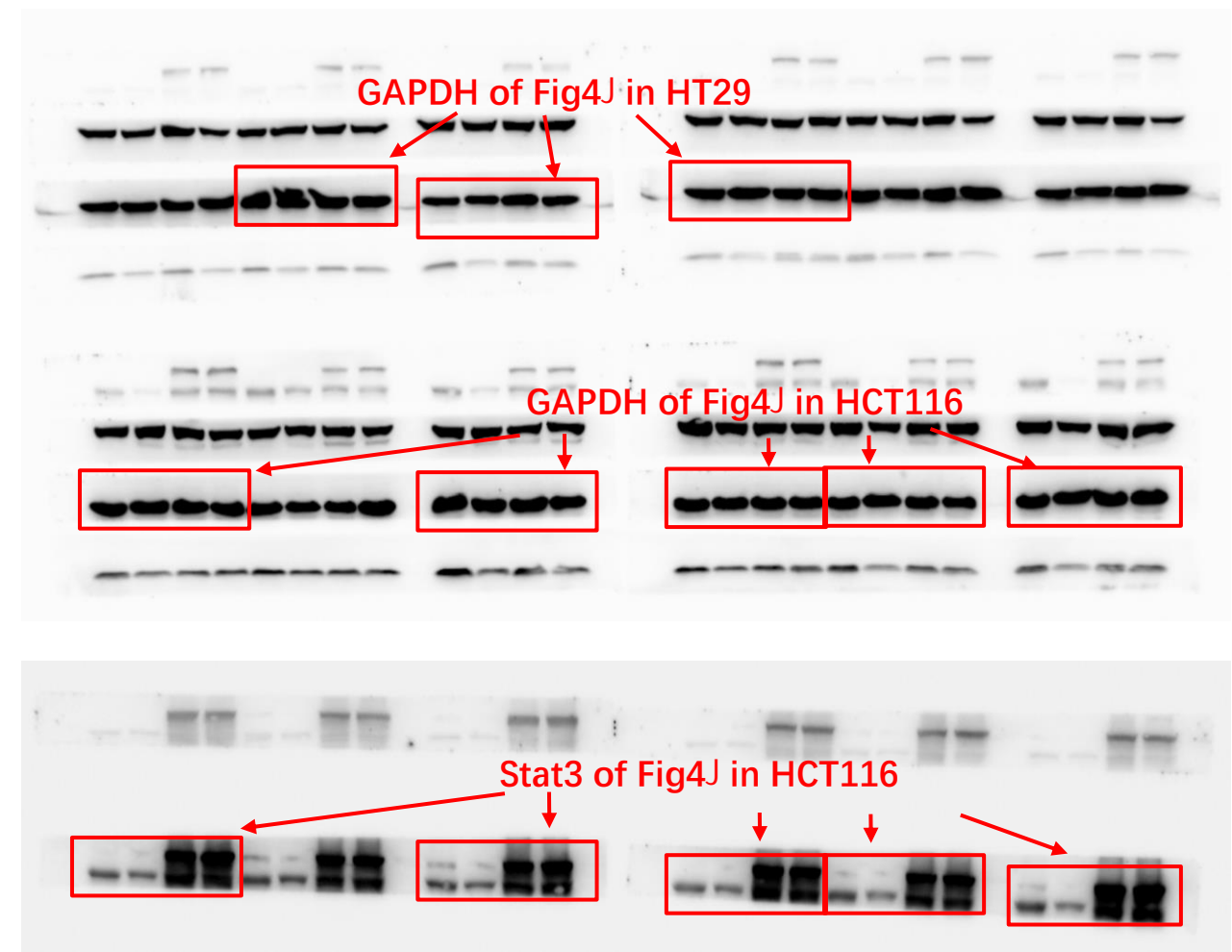

Fig 5C

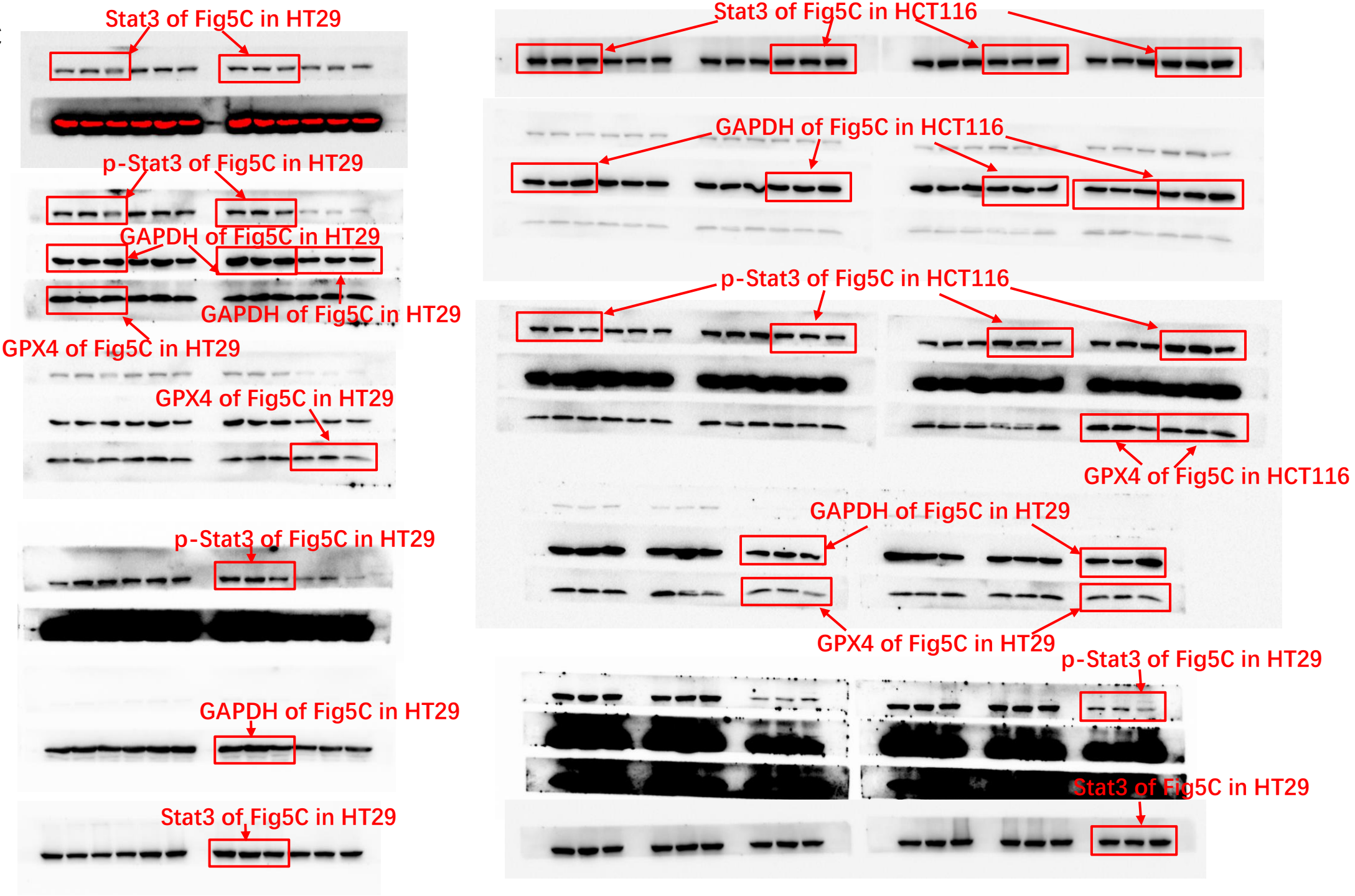

Fig 5C

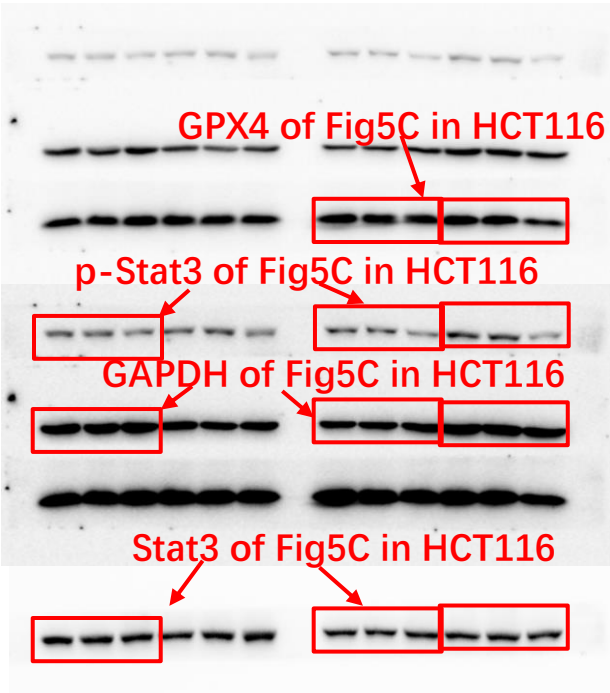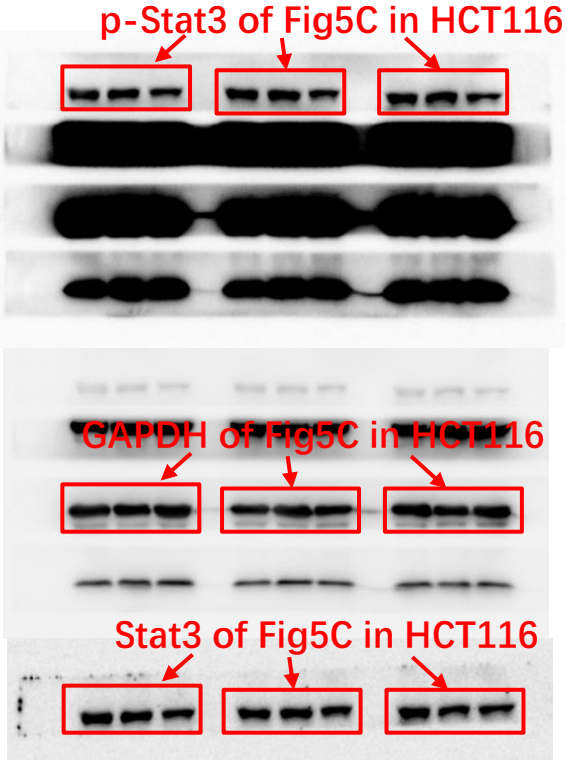

Fig 6G

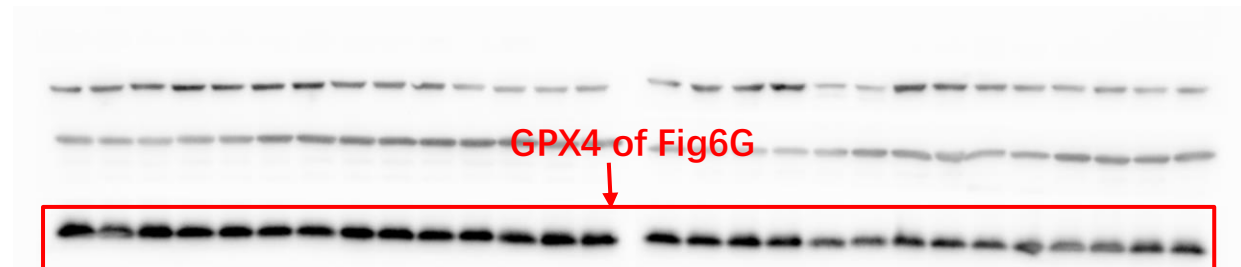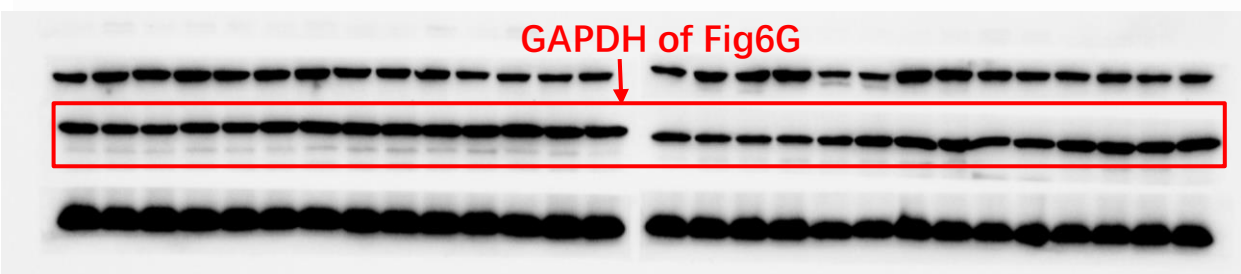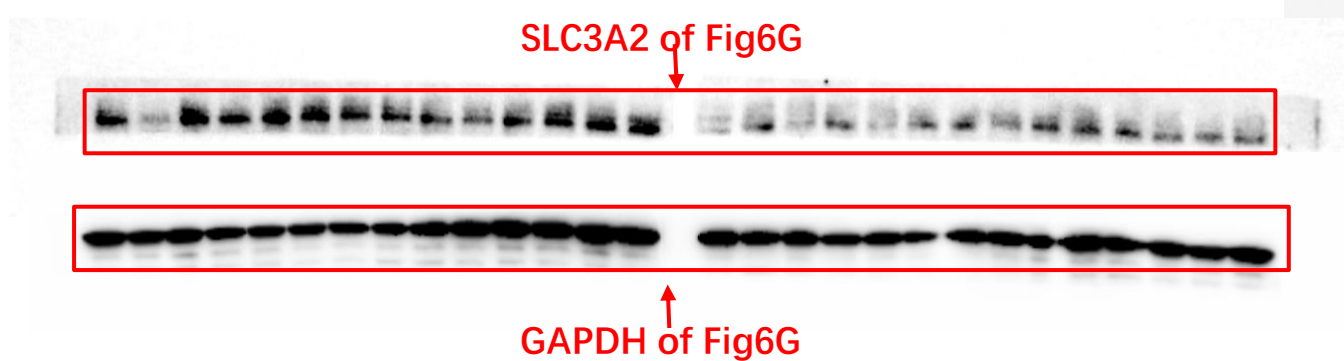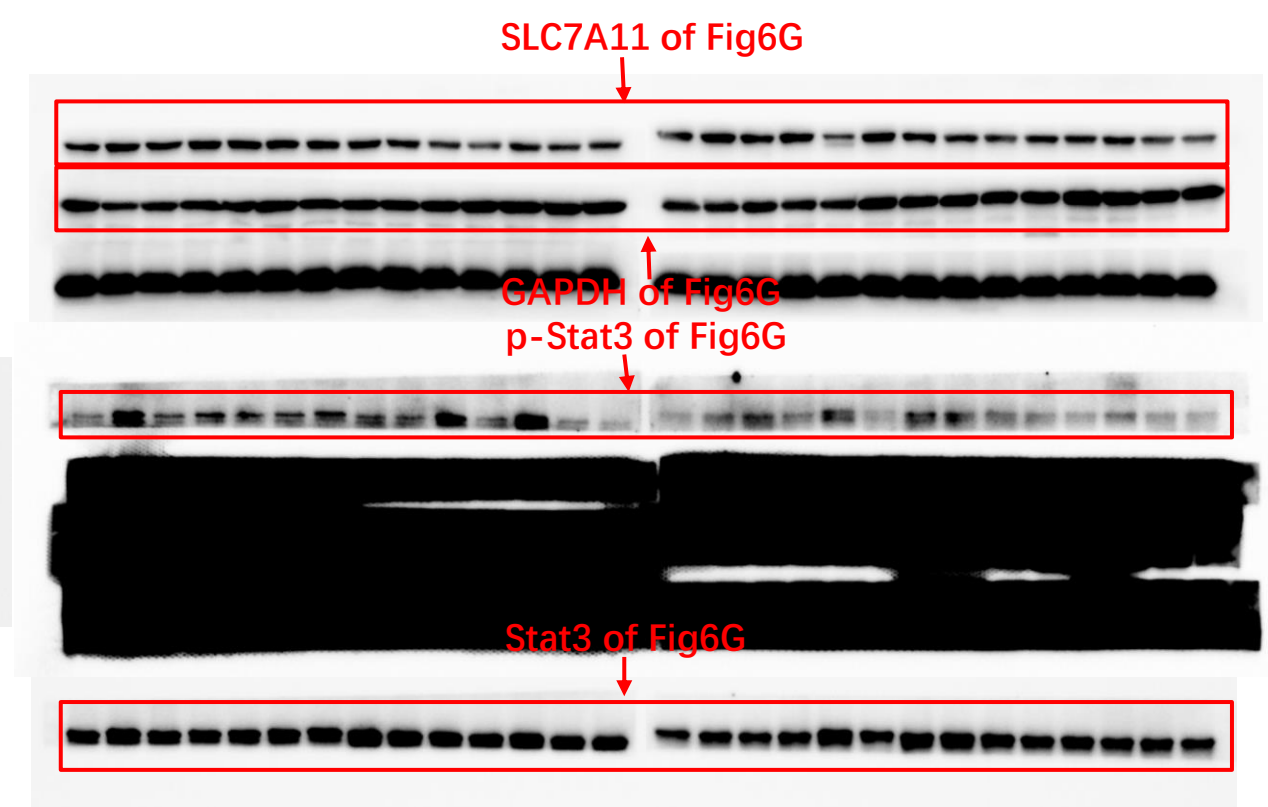

Supplement: Supplementary file 2 [file DataSheet1.pdf]
